# *Haemophilus pittmaniae* and *Leptotrichia* spp. Constitute a Multi-Marker Signature in a Cohort of Human Papillomavirus-Positive Head and Neck Cancer Patients

**DOI:** 10.3389/fmicb.2021.794546

**Published:** 2022-01-18

**Authors:** Jean-Luc C. Mougeot, Micaela F. Beckman, Holden C. Langdon, Rajesh V. Lalla, Michael T. Brennan, Farah K. Bahrani Mougeot

**Affiliations:** ^1^Carolinas Medical Center—Atrium Health, Charlotte, NC, United States; ^2^Section of Oral Medicine–University of Connecticut Health, Farmington, CT, United States

**Keywords:** head and neck cancer, HPV, oral microbiome, next generation sequencing, *Leptotrichia* spp.

## Abstract

**Objectives:**

Human papillomavirus (HPV) is a known etiological factor of oropharyngeal head and neck cancer (HNC). HPV positivity and periodontal disease have been associated with higher HNC risk, suggesting a role for oral bacterial species. Our objective was to determine oral microbiome profiles in HNC patients (HPV-positive and HPV-negative) and in healthy controls (HC).

**Methods:**

Saliva samples and swabs of buccal mucosa, supragingival plaque, and tongue were collected from HNC patients (*N* = 23 patients, *n* = 92 samples) before cancer therapy. Next-generation sequencing (16S-rRNA gene V3–V4 region) was used to determine bacterial taxa relative abundance (RA). β-Diversities of HNC HPV+ (*N* = 16 patients, *n* = 64 samples) and HNC HPV– (*N* = 7 patients, *n* = 28 samples) groups were compared using PERMANOVA (pMonte Carlo < 0.05). LEfSe discriminant analysis was performed to identify differentiating taxa (Log LDA > 2.0). RA differences were analyzed by Mann–Whitney *U*-test (α = 0.05). CombiROC program was used to determine multi-marker bacterial signatures. The Microbial Interaction Network Database (MIND) and LitSuggest online tools were used for complementary analyses.

**Results:**

HNC vs. HC and HNC HPV+ vs. HNC HPV– β-diversities differed significantly (pMonte Carlo < 0.05). *Streptococcus* was the most abundant genus for HNC and HC groups, while *Rothia mucilaginosa* and *Haemophilus parainfluenzae* were the most abundant species in HNC and HC patients, respectively, regardless of antibiotics treatment. LEfSe analysis identified 43 and 44 distinctive species for HNC HPV+ and HNC HPV– groups, respectively. In HNC HPV+ group, 26 periodontal disease-associated species identified by LefSe had a higher average RA compared to HNC HPV– group. The significant species included *Alloprevotella tannerae*, *Fusobacterium periodonticum*, *Haemophilus pittmaniae*, *Lachnoanaerobaulum orale*, and *Leptotrichia* spp. (Mann–Whitney *U*-test, *p* < 0.05). Of 43 LEfSe-identified species in HPV+ group, 31 had a higher RA compared to HPV– group (Mann–Whitney *U*-test, *p* < 0.05). MIND analysis confirmed interactions between *Haemophilus* and *Leptotrichia* spp., representing a multi-marker signature per CombiROC analysis [area under the curve (AUC) > 0.9]. LitSuggest correctly classified 15 articles relevant to oral microbiome and HPV status.

**Conclusion:**

Oral microbiome profiles of HNC HPV+ and HNC HPV– patients differed significantly regarding periodontal-associated species. Our results suggest that oral bacterial species (e.g., *Leptotrichia* spp.), possessing unique niches and invasive properties, coexist with HPV within HPV-induced oral lesions in HNC patients. Further investigation into host–microbe interactions in HPV-positive HNC patients may shed light into cancer development.

## Introduction

Head and neck cancer (HNC) is the sixth most common cancer worldwide with over 95% comprising squamous cell carcinomas (SCCs) (Jemal et al., 2008; Kumarasamy et al., 2019). Head and neck SCCs are characterized by a locoregional development mainly diagnosed at an advanced stage of the disease, resulting in difficult treatment and eradication of both pre-neoplastic and neoplastic tissue (Carvalho et al., 2005; Ganci et al., 2015). Despite advancements in chemoradiation, ionizing radiation, and surgical resection techniques, HNC has an overall mortality rate of approximately 50% and is characterized by high recurrence rates (Carvalho et al., 2005). While triggers of HNC development have not been fully elucidated, two primary risk factors have been identified, namely, alcohol and tobacco consumptions (Božinović et al., 2019). Most recent studies have identified infection with human papillomavirus (HPV) as a third and more prominent cause of tumor formation (Božinović et al., 2019). HPV-associated SCCs represent the most common HPV-related cancer in the US and are classified by a new staging system for oropharyngeal cancers (National Cancer Institute^1^ : van Gysen et al., 2019).

HPV-positive (HPV+) HNC patients are often younger and present with a more advanced cancer stage than HPV-negative (HPV–) HNC patients (Blitzer et al., 2014). It has been reported that the majority of HPV-associated HNCs are caused by HPV16, though more than 220 HPV serotypes have been identified (Tumban, 2019). Two HPV genes, E6 and E7, have been the matter of extensive research due to their role as oncogenes (Yim and Park, 2005). These genes are involved in multiple pathways such as transmembrane signaling, cell cycle regulation, and cell transformation (Yim and Park, 2005). E6 has been shown to promote degradation of tumor suppressor TP53 (Crook et al., 1991), while E7 is able to inhibit retinoblastoma protein (Pal and Kundu, 2020). Aside from E6 and E7, the E5 HPV gene promotes malignancy, has anti-apoptotic effects and plays a role in epidermal growth factor (EGF) receptor-regulated cell proliferation (Venuti et al., 2011).

There is a mounting body of evidence that a synergistic interaction between periodontal disease-associated pathogens and HPV exists. Indeed, a case–control study by Tezal et al. (2009) found that HPV+ tumors in 21 patients had a significantly higher alveolar bone loss mean and a fourfold increased risk for HPV+ tumor status for every millimeter of alveolar bone loss caused by periodontal disease.

Overall, without implying a causal effect, a link between oral microbiome dysbiosis and cancer has been suggested by several studies (Kudo et al., 2016; Hayes et al., 2018; Mohammed et al., 2018; Wu et al., 2018; Mougeot et al., 2020). For instance, *Fusobacterium nucleatum* was found overabundant in the oral cavity of patients with colon cancer and lymph node metastasis (Kudo et al., 2016). *F. nucleatum* might initiate oncogenic and proinflammatory responses that stimulate the growth of colon cancer cells (Kudo et al., 2016). Increased levels of blood serum antibodies against the oral bacterial species *Porphyromonas gingivalis* was associated with a twofold higher risk of pancreatic cancer when compared to healthy individuals (Mohammed et al., 2018).

Furthermore, the increased prevalence of *P. gingivalis* and *Aggregatibacter actinomycetemcomitans* was shown to initiate a Toll-like receptor signaling pathway predictive of pancreatic cancer in animal models (Mohammed et al., 2018). Higher levels of firmicutes and bacteroidetes also constitute a potential risk for gastric cancer (Wu et al., 2018). A study by Hayes et al. (2018) suggested an association between the oral microbiome and HNC.

HNC has also been associated with oral cavity diseases such as periodontal disease and dental caries (Michaud et al., 2017; Gasparoni et al., 2021). Periodontitis disrupts the normal oral microbial environment, thereby leading to dysbiosis. Dysbiosis can translate into an abundance shift of opportunistic species, like *P. gingivalis*, which produce several virulence factors resulting in the destruction of periodontal tissues (Rafiei et al., 2017). Chronic periodontitis can result in the release of proinflammatory cytokines from squamous cells, causing inflammation and possible decreased apoptosis (Gholizadeh et al., 2016). In a meta-analysis by Zeng et al. (2013) HNC cancer risk was found to be increased by 2.63-fold in patients with periodontitis.

Using 16S rRNA gene next-generation sequencing and computational approaches, the purpose of this study was to compare microbiome profiles of a limited cohort of HNC patients to those of healthy control subjects, and profiles of HNC HPV+ patients to those of HNC HPV– patients, within the HNC cohort. We also aimed to determine whether bacterial species differentiating HPV+ from HPV– HNC patients are associated with periodontal disease-associated species.

## Materials and Methods

### Patient Recruitment

HNC patients with SCC [*N* = 30; 8 females, 22 males, age range = 23–75 years (SD = ± 12.02)] were recruited from the OraRad study (U01DE022939) (Brennan et al., 2017; Lalla et al., 2017). OraRad was a multicenter cohort study that collected longitudinal data on radiation-treated HNC patients at 6-month intervals for 2 years. Primary cancer site origin included the base of the tongue, tonsil, neck, tongue, and oral cavity.

Of 30 HNC patients, 23 were clinically classified as HPV+/− into 16 HPV+ and 7 HPV– patients. In addition, healthy control subjects (HC group) (*N* = 20; age range 24–84, *SD* = –12.93) were recruited through Atrium Health’s Carolinas Medical Center, Charlotte, NC. Of 30 HNC patients, 11 had received antibiotic treatment within 2 weeks of sampling. No HC subject had received antibiotic treatment. The study was approved by the institutional review board, and all participants gave informed consent for the study.

### Sample Collection

Saliva (S) samples and swab samples of buccal mucosa (B), supragingival plaque (P), and tongue (T) were collected from HNC patients, pre-cancer treatment at baseline, and from HC subjects. Saliva collection was performed while chewing unsweetened and unflavored gum (The Wrigley Company—Mars, Chicago, IL, United States) for a period of 2 min into a 50-ml conical BD falcon polypropylene centrifuge tube (Corning, Corning, NY, United States).

Buccal mucosal samples were subsequently collected by swabbing both sides of the buccal mucosa for 10 s each. Tongue samples were then obtained by swabbing a 1-cm^2^ region on both sides of the mid-dorsal region of the tongue for 5 s. Finally, supragingival plaque samples were obtained by swabbing across the lateral surfaces of all maxillary and mandibular teeth at the junction of the tooth and gingiva. All swab collections were performed using OmniSwabs (GE Life Sciences-Buckinghamshire, United Kingdom).

### Bacterial DNA Extraction, Processing, and Sequencing

Bacterial genomic DNA was extracted from oral samples using QIAamp DNA Mini Kit procedure (QIAGEN, Valencia, CA, United States) per manufacturers’ instructions. During sample preparation, 50 ng of genomic DNA was used for PCR in which the 16S rRNA gene (V3–V4) region was amplified, followed by purification and processing methods as previously described (Caporaso et al., 2011). Next-generation sequencing was performed using the MiSeq v3 reagent kit and platform (Illumina, Inc., San Diego, CA, United States). To prepare for cluster generation and sequencing, libraries were denatured with NaOH and diluted with a hybridization buffer. Libraries then underwent heat denaturation prior to MiSeq sequencing. Total of 100 ng of each library was pooled together, run on a gel, gel-extracted, and run on a bioanalyzer for quantification. A total concentration of 4 nM of the library was then diluted, and 12 pM of the library was spiked with 20% PhiX. At least 5% PhiX was added as an internal control for low-diversity libraries. Identification of bacterial genera and species was performed using Human Oral Microbe Identification, HOMI*NGS*, which employs a ProbeSeq BLAST program for species/genera identification through recognition of the 16S rRNA gene (V3–V4 region) sequence reads (Caporaso et al., 2011; Mougeot et al., 2016). ProbeSeq loads raw sequence files into a cell array, looping through the array one sequence at a time searching for small sequence strings that 100% match an oligomer (partials are not considered matches). If a match is identified, a counter begins giving counts of the total number of probe-specific “hits.” Hits are then accumulated by species/genera and sample.

The sequence reads were matched to 737 ProbeSeq taxon probes, i.e., to species probes (*n* = 620) or genus probes (*n* = 117) if not matched to a species probe, or were otherwise recorded as an unmatched read. Matched and unmatched probe count data were provided per taxon per patient as Excel spreadsheets. Species/genus probes containing zeros for all samples were removed from the dataset. Raw abundance data were then transformed into relative abundance (RA) data for further analysis.

### Bioinformatic Analysis

#### α-Diversity

Shannon and Simpson indices were generated using PRIMER**_v7_** (PRIMER-E Ltd., Ivybridge, United Kingdom) (Clarke and Gorley, 2006), based on microbiome RA data. RA data of HC subjects (HC group: *N* = 20) were compared to the RA data of HNC patients including those with antibiotic treatment within 2 weeks of sampling (Grp-All: *N* = 30). RA data of HC group were also compared to RA data of HNC patients excluding those with antibiotic treatment (Grp-NoAB: *N* = 19). Subsequently, comparisons of HPV+ vs. HPV– HNC sub-cohorts were performed by including or excluding patients who received antibiotic treatment. Mann–Whitney *U*-tests were then used to determine significant RA comparisons (α = 0.05) using XLSTAT**_v2016.02.29253_** (Data Analysis and Statistical Solution for Microsoft Excel, Addinsoft, Paris, France, 2017).

#### Permutational Multivariate Analysis of Variance

**FIGURE 1:**
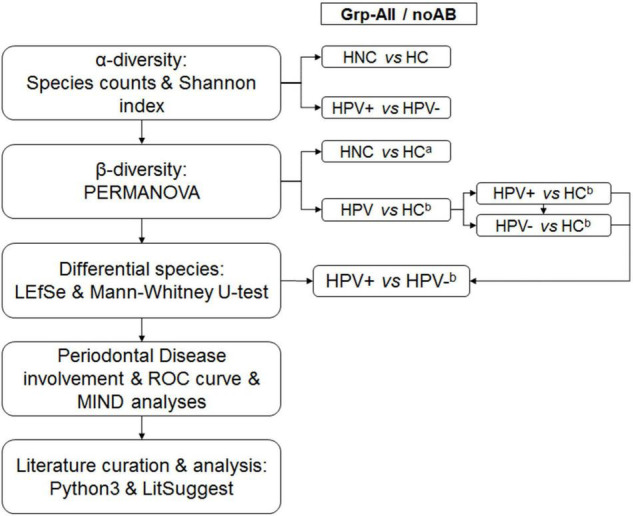
Overall analytical strategy. α-Diversity was calculated for Grp-All HNC vs. HC (*N* = 30; *N* = 20) and HPV+ vs. HPV– (*N* = 16; *N* = 7) and for Grp-noAB HNC vs. HC (*N* = 19; *N* = 20) and HPV+ vs. HPV– (*N* = 12; *N* = 3). Sample sites consisted of different combinations of buccal (B), plaque (P), saliva (S), and tongue (T) samples. β-Diversity was calculated using PERMANOVA by two different analytical designs: (1) *^a^*Analytical design with “Diagnosis” and “Sample site” as fixed factors and “Antibiotic” as the nested random factor. Comparisons were Grp-All HNC vs. HC and Grp-noAB HNC vs. HC. (2) *^b^*Analytical design with “HPV status” and “Sample site” as fixed factors and “Antibiotic” as the nested random factor. Grp-All comparisons were as follows: HPV vs. HC (*N* = 23; *N* = 20), HPV+ vs. HC (*N* = 16; *N* = 20), HPV– vs. HC (*N* = 7; *N* = 20), and HPV+ vs. HPV– (*N* = 16; *N* = 7). Grp-noAB comparisons were as follows: HPV vs. HC (*N* = 15; *N* = 20), HPV+ vs. HC (*N* = 12; *N* = 20), HPV– vs. HC (*N* = 3; *N* = 20), and HPV+ vs. HPV– (*N* = 12; *N* = 3). Distinct species were determined using LEfSe and Mann–Whitney *U*-tests for Grp-All and Grp-noAB comparisons of HPV+ vs. HPV– for the sample site combination BPST. Significant LEfSe bacterial species for HPV+ group were investigated for their roles in periodontal disease using conventional methods (PubMed, Google Scholar, etc.). Significant LEfSe HPV+ species using the Mann–Whitney *U*-test were analyzed by using MedCalc ROC curve analysis. Multi-marker combinatorial analysis was completed using CombiROC online tool (http://combiroc.eu/) for the bacterial species identified with MedCalc log(RA + 1) ROC curve AUC of 0.75 or greater (*n* = 10 species). CombiROC utilized raw RA data and log(RA + 1) transformed data. Microbial Interaction Network Database (MIND) online tool was used to identify possible interactions between most significant bacterial species. Python_v3.6.2_ code was used to extract PubMed abstracts matching key words to determine positive and negative training sets and were validated manually. Using NCBI LitSuggest online tool (https://www.ncbi.nlm.nih.gov/research/litsuggest/), 203 PubMed articles were classified to identify articles involving HPV infection in the oral microbiome.

Patient subgroups used for permutational multivariate analysis of variance (PERMANOVA) included Grp-All (HNC: *N* = 30; HC: *N* = 20) and Grp-noAB (HNC: *N* = 19; HC: *N* = 20). Sub-analyses were performed based on the multiple sample site combinations “BPST,” “BST,” and “PST” which provided sufficient power in PRIMER**_v7_** program (PRIMER-E Ltd., Ivybridge, United Kingdom) (Clarke and Gorley, 2006) for all five relevant comparisons (Figure 1). Species and genera RA data were square root transformed and converted into Bray–Curtis similarity matrices.

PERMANOVA analyses were performed using a mixed model with unrestricted permutation of raw data, 9,999 permutations, and type III partial sum of squares (Clarke and Gorley, 2006), as previously implemented (Mougeot et al., 2019, 2020). Fixed factors were “Diagnosis” (e.g., HNC vs. HC) and “Sample site” (B, P, S, and T). In this design, the “Antibiotic” treatment (yes or no) variable was used as a random factor nested into “Diagnosis” and “Sample site.” Monte Carlo corrected *p*-values (α = 0.05) were determined, as appropriate for relatively small sample sizes. Principal coordinate analysis (PCoA) was completed for the Grp-All: HNC vs. HC BPST sample site combination.

#### β Diversity Sub-Analyses

Sub-analyses were completed using subsets of Grp-All and Grp-noAB patients, based on *n* = 3 or 4 sample sites per patient (BPST, BST, and PST): HPV+ (*N* = 16) vs. HPV– (*N* = 7) and HPV+ (*N* = 12) vs. HPV– (*N* = 3), respectively. The sample site combinations BPST, BST, and PST and the previously mentioned data transformation were used for PERMANOVAs in PRIMER**_v7_** program. Fixed factors used were “HPV status” (positive and negative) and “Sample site” (B, P, S, and T). “Antibiotic” (yes or no) was used as a random factor and nested into “HPV status” and “Sample site.” Monte Carlo corrected *p*-values (α = 0.05) were determined. PCoA was completed for the Grp-All: HPV+ vs. HPV– BPST sample site combination.

#### Linear Discriminant Analysis Effect Size

Taxonomy levels were added manually to ProbeSeq derived datasets for Grp-All (HPV+ vs. HPV–) and Grp-noAB (HPV+ vs. HPV–) subsets. The text files were then formatted for linear discriminant analysis (LDA) effect size (LEfSe) using the Galaxy**_v1.0_** online tool (Jalili et al., 2020). LEfSe data input consisted of “HPV status” as the option “Class” and “Patient ID” as the option “Subject” (Segata et al., 2011). Data were normalized. Using the “one-against-all” strategy for multi-class analysis, the factorial Kruskal–Wallis test and pairwise Wilcoxon signed-rank test were set at a Monte Carlo significance (α = 0.05) to calculate LDA scores. Log LDA scores were set at a threshold > 2.0. Histograms of the differential features (species) were generated, and each species was investigated for its role in periodontal disease.

### Receiver-Operating Characteristic Curve Analyses

#### Conventional Receiver-Operating Characteristic Analysis

Mann–Whitney *U*-tests were completed for LEfSe differential features for HPV+ species probes from Grp-All and Grp-noAB groups. Significant species probes (α = 0.05) further underwent receiver-operating characteristic (ROC) curve analysis for Grp-All HPV+ and Grp-noAB HPV+ species probes using the BPST sample combination in MedCalc program (MedCalc Software Ltd, Ostend, Belgium).

RA data were log-transformed with the addition of a pseudo-count [i.e., log(RA + 1)]. Analysis was completed for Grp-All (HPV+; *n* = 64 samples and HPV–; *n* = 28 samples) and Grp-noAB (HPV+; *n* = 48 samples and HPV–; *n* = 12 samples) groups and for each non-zero RA probe in MedCalc program. The area under the curve (AUC) of each probe was calculated, and ROC curves were generated. Significance level was set at α = 0.05, and biomarker accuracy was calculated using methods described by Ray et al. (2010).

#### CombiROC Analysis

ROC curves from MedCalc that had an AUC greater than 0.75 were subjected to combinatorial analysis using CombiROC online tool^2^ (Mazzara et al., 2017) based on raw RA data and log(RA + 1) transformed RA data. Using CombiROC, marker profile plots were generated to confirm quality, and the detection threshold was set to 0.001. Using this threshold, combinational analysis was performed which calculated the sensitivity and specificity scores for each marker or combination of markers corresponding to the probability that the microbial data will be positive when HPV is present and the probability that microbial data will be negative when HPV is not present.

A minimum feature filter was set to include at least two markers. Based on a threshold of 10 for sensitivity and 50 for specificity, the best or “gold” combinations of markers were kept, thereby creating optimal multi-marker ROC curves and violin plots. Summary statistics were calculated and recorded for the top two AUC scores of the raw and log(RA + 1) transformed RA data.

### Microbial Interaction Network Database Analysis

A microbial interaction network was created to illustrate possible interactions between *Haemophilus* spp. and *Leptotrichia* spp. with other bacterial genera or species, by using Microbial Interaction Network Database (MIND**_v1.0_**) (Microbial Interaction Network Database, 2019). Default options were selected for human tissue sites, interaction weight, and health or disease conditions.

### LitSuggest

An application programming interface was established using the National Center for Biotechnology Information guidelines (National Center for Biotechnology Information, 1988) and Python**_v3.6.2_** (van Rossum and Drake, 2009). Python**_v3.6.2_** was used to generate classifiers by extracting abstracts from PubMed (1997) through the keywords (i) “oral microbiome” and “HPV” to constitute a positive training set, (ii) “vaginal microbiome” and “HPV” keywords to constitute the negative training set, and (iii) “HPV” and “microbiome” to constitute the test set. Positive and negative training set abstracts were then manually validated. Using the NCBI LitSuggest^3^ online tool; a total of 19 positively and 104 negatively classified articles were used to train the model (Allot et al., 2021). Test set classification was then completed using LitSuggest, and full articles were manually verified for relevancy.

## Results

The overall analytical strategy is presented in Figure 1. Demographics and clinical information of our HNC patient cohort (*N* = 30 patients, sub-cohort of OraRad study) are presented in Table 1. Clinical information, including caries and periodontal disease status for OraRad HNC patient cohort, associated with HPV status (*N* = 559 of 572 total patients) has been published elsewhere (Brennan et al., 2021). While no significant differences were noted in age and ethnicity, the male population was over-represented in the HNC patient set in OraRad and this study, as anticipated for oral SCC in general and for HPV-associated oropharyngeal cancers (Fakhry et al., 2018; Mahal et al., 2019). In our sub-cohort, most HPV+ HNC patients had oropharyngeal cancer (e.g., tonsil, base of tongue), whereas most of the HPV– HNC patients had cancer in other sites (Table 1).

**TABLE 1:** Patient demographics and clinical characteristics.

	HNC^a^	HC^b^	Combined^c^	HNC HPV+^d^	HNC HPV−^e^	Combined^f^
**Patient count (Male/Female)**	30 (22/8)	20 (5/15)	50 (27/23)	16 (12/4)	7 (5/2)	23 (17/6)
**Antibiotic treatment (Yes/No)**	11/19	0/20	11/39	4/12	4/3	8/15
**Primary cancer site**						
Base of tongue	4		4	4	0	4
Nasopharynx	2		2	1	1	2
Oral cavity	1		1	0	1	1
Oropharynx	1		1	1	0	1
Supraglottis	1		1	0	1	1
Tongue	1		1	0	1	1
Tonsil	8		8	8	0	8
Unknown	5		5	2	3	5
**Age:**						
Median	55	55	55	54	61	54
Mean	54	52.7	54	54	51	53
Std Dev	12.02	15.29	12.93	6.47	20.29	11.93
Range	23–75	24–84	23–84	40–68	23–75	23–75
**Ethnicity count**						
M: Caucasian/African American	22/0	5/0	27/0	12/0	5/0	17/0
F: Caucasian/African American	7/1	13/2	20/3	3/1	2/0	5/1
**Whole mouth average PD**				2.28 (2.03–2.52)	2.09 (1.78–2.40)	2.26 (2.06–2.46)
**Whole mouth average CAL**				1.74 (1.42–2.07)	1.64 (1.02–2.26)	1.73 (1.46–2.01)
**Sample combinations*^g^***						
BPST	120	80	200	64	28	92
BST	120	72	192	54	30	84
PST	105	69	174	54	21	75
BPS	93	63	156			

*^a^Head and neck cancer (HNC) patient group (primary cancer sites: base of tongue = 4; nasopharynx = 2; oral cavity = 1; oropharynx = 1; supraglottis = 1; tongue = 1; tonsil = 8; unknown = 5).*

*^b^Healthy control (HC) subject group.*

*^c^HNC and HC patient groups combined.*

*^d^HNC human papillomavirus positive (HPV+) patient group (primary cancer sites (N = 16); base of tongue = 4; nasopharynx = 1; oral cavity = 0; oropharynx = 1; supraglottis = 0; tongue = 0; tonsil = 8; unknown = 2).*

*^e^HNC human papillomavirus negative (HPV–) patient group (primary cancer sites (N = 7); base of tongue = 0; nasopharynx = 1; oral cavity = 1; oropharynx = 0; supraglottis = 1; tongue = 1; tonsil = 0; unknown = 3).*

*^f^HNC HPV+ and HPV– patient groups combined.*

*^g^Number of samples for site combinations including B (buccal), P (plaque), S (saliva), and/or T (tongue).*

*PD, probing depth; CAL, clinical attachment loss; Std Dev, standard deviation. PD and CAL are shown as the average with 95% confidence intervals in parentheses. Mann–Whitney U-tests comparing whole mouth average PD and whole mouth average CAL separately for HPV+ vs. HPV– patient groups were not found to be significantly different (p > 0.05).*

### Abundance, Species Detection, and α-Diversity

Probe count data are provided as Supplementary Data Files and can be downloaded from our lab’s Github repository^4^ (Supplementary Data Files 1, 2). Sequencing reads matched 737 total probes (117 genera and 620 species probes) for all samples from HNC and HC groups combined. Comparisons of species and genera detected for HNC vs. HC and HPV+ vs. HPV– are presented in Supplementary Table 1. Unmatched reads were removed from RA determinations. For all samples sequencing data, 442 of 620 species probes and 65 of 117 genus probes had at least one matched read. Significant α-diversity differences were identified for Grp-All and Grp-noAB for HNC vs. HC and Grp-noAB HNC vs. HC for the matched sample site combinations BPST, BST, and PST (Table 2). *Streptococcus* was the most abundant genus for HNC and HC groups, whereas *Rothia mucilaginosa* and *Haemophilus parainfluenzae* were the most abundant species detected in HNC patients and HC, respectively. Excluding HNC patients treated with antibiotics (Grp-noAB) did not affect these results (data not shown). Overall, the highest and lowest average number of taxa detected per sample were 96.08 and 117.66 for species probes and 24.75 and 26.83 for genus probes (Supplementary Table 1).

**TABLE 2:** α-diversity comparisons: HNC vs. HC and HNC HPV+ vs. HPV–.

Variable^a^	Min^b^	Max^c^	Mean^d^	Std Dev^e^	*p*-value^f^
**Grp-All HNC vs. HC**					
**BPST**					
HNC	42	247	131.65	39.738	**0.045**
HC	53	281	143.5	42.448	
**BST**					
HNC	42	224	127.87	36.689	**0.004**
HC	72	281	145.96	41.343	
**BPS**					
HNC	45	247	138.28	42.348	0.378
HC	53	581	143.91	44.884	
**PST**					
HNC	42	267	135.09	42.93	**0.027**
HC	53	223	146.37	38.401	
**Grp-noAB HNC vs. HC**					
**BPST**					
HNC	45	247	131.17	38.434	0.056
HC	53	281	143.5	42448	
**BST**					
HNC	45	224	139.91	33.5	**0.017**
HC	72	281	145.96	41.343	
**BPS**					
HNC	45	247	139.55	41.855	0.54
HC	53	281	14.91	44.884	
**PST**					
HNC	69	267	136.13	42.264	**0.045**
HC	53	223	146.38	38.401	
**Grp-All HPV+ vs. HPV−**					
**BPST**					
Negative	42	218	127.25	37.99	0.413
Positive	45	247	133.47	39.39	
**BST**					
Negative	42	218	119.67	40.014	0.063
Positive	45	214	134.20	34.948	
**PST**					
Negative	42	218	130.52	40.964	0.483
Positive	69	267	140.30	43.242	
**Grp-noAB HPV+ vs. HPV−**					
**BPST**					
Negative	86	177	128.83	28.197	0.919
Positive	45	247	130.17	40.511	
**BST**					
Negative	86	174	128.08	28.273	0.787
Positive	45	214	130.71	34.437	
**PST**					
Negative	86	177	133.67	30.332	0.921
Positive	69	267	137.29	45.687	

*^a^Sample comparisons from head and neck cancer (HNC) patients with or without human papillomavirus (HPV) and healthy controls (HC) for sample sites buccal (B), plaque (P), saliva (S), and tongue (T) with and without (noAB) antibiotic treatment.*

*^b^Minimum number of species detected per sample.*

*^c^Maximum number of species detected per sample.*

*^d^Mean number of species detected per sample.*

*^e^Standard deviation of species detected per sample.*

*^f^Mann–Whitney U-test p-value.*

*Significant values (p > 0.05) are shown in bold. Positive, HPV positive; Negative, HPV negative.*

### β-Diversity Analysis

**FIGURE 2:**
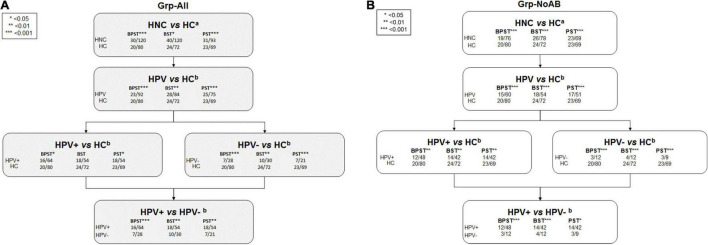
PERMANOVA results comparisons flowchart. **(A)** Grp-All. **(B)** Grp-noAB. β-diversity analyses were performed using PERMANOVA in PRIMER_v7_ software (PRIMER-E Ltd., IvyBridge, United Kingdom) to compare microbial profiles of head and neck cancer (HNC) patients to healthy controls (HC) and to compare microbial profiles of HNC HPV-positive (HPV+) patients to HNC HPV– patients. Sample sites consisted of three to four site combinations of buccal (B), plaque (P), saliva (S), and tongue (T). PERMANOVA analysis was completed using two different analytical designs based on Bray–Curtis similarity matrices determined from square root transformed relative abundance data of 737 probes (620 species and 117 genus probes). Sample site combinations consisted of BPST, BST, and PST for **(A)** Grp-All HNC vs. HC (*N* = 30; *N* = 20) and **(B)** Grp-no Antibiotics (Grp-noAB) HNC vs. HC (*N* = 19; *N* = 20). *^a^*For the Grp-All HNC vs. HC comparison, “Diagnosis” was the main fixed factor, and “Sample Site” was the secondary fixed factor. “Antibiotics” was nested into “Diagnosis” and “Sample site” factors as a random variable. Grp-noAB analytical design did not include antibiotics as a factor. *^b^*For the analytical design considering HPV, “HPV status” and “Sample site” were set as fixed factors and “Antibiotics” as nested as random factor. Grp-noAB analytical design did not include antibiotics as a factor. Level of significance is denoted using an asterisk (*): * < 0.05 = *p*-value less than 0.05; ** < 0.01 = *p*-value less than 0.01; *** < 0.001 = *p*-value less than 0.001.

PERMANOVA β-diversity analyses were performed for sample site combinations providing sufficient power based on available oral microbiome data (i.e., BPST, BST, and PST). Significance of β-diversity analyses is presented in Figure 2. For the Grp-All comparisons, including HNC HPV+ vs. HNC HPV– comparisons, all but one (i.e., HPV+ vs. HC, BST, pMonte Carlo = 0.261) were significant, regardless of the sample site combinations analyzed. All Grp-noAB comparisons were found significant for “HPV status” and “Sample site.” Monte Carlo corrected *p-*values of all comparisons are presented in Supplementary Table 2. PCoA plots describing the variations explaining dissimilarity between groups (i.e., HNC vs. HC and HPV+ vs. HPV–) are presented in Supplementary Figure 1.

### LEfSe Analysis

**FIGURE 3:**
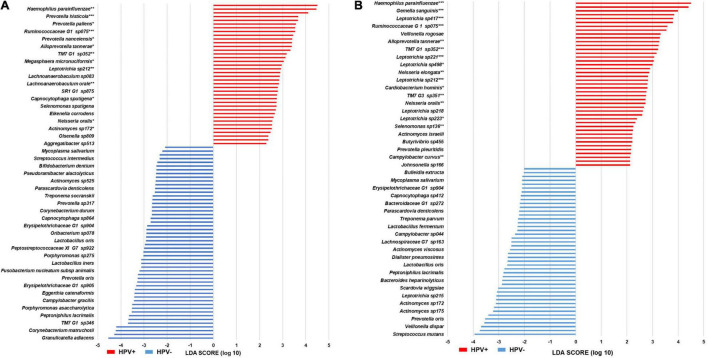
LEfSe histograms of differential features in head and neck cancer patients. **(A)** Grp-All BPST. **(B)** Grp-noAB BPST. Linear discriminant analysis Effect Size (LEfSe) was performed to determine distinct microbiome features in oral samples [stimulated saliva (S) samples and swabs of buccal mucosa (B), supragingival plaque (P), and tongue (T)] of the following: **(A)** Grp-All patient cohort (*N* = 23) for the head and neck cancer (HNC) HPV-positive (HPV+; *N* = 16) vs. HPV-negative (HPV–; *N* = 7) comparisons. **(B)** Grp-noAB patient cohort (*N* = 15) for HNC HPV+ (*N* = 12) vs. HPV– (*N* = 3) that did not receive antibiotics within 2 weeks of sampling. Horizontal histograms depict the discriminant features, i.e., bacterial species, for Grp-All and Grp-noAB potential biomarkers for HPV+ (red) and HPV– (blue). Mann–Whitney *U*-tests were used to determine significance of HPV+ distinctive species. Level of significance is depicted by an asterisk (*): *p < 0.05; **p < 0.01; ***p < 0.001.

A total of 44 and 43 species were identified for Grp-All HPV– and HPV+, respectively. A histogram of the differential features is presented in Figure 3A. Species of the genera *Actinomyces* and *Leptotrichia* were the most representative of HPV– and HPV+ patient groups, respectively. A total of 52 and 38 species were identified for Grp-noAB HPV– and HPV+, respectively (Figure 3B). *Leptotrichia* spp. were the most represented taxa for Grp-noAB HPV+ patients, and *Prevotella* spp. were the most represented ones for Grp-noAB HPV– patients. A total of 26 of 43 species in Grp-All HPV+ group (60.5%) and 24 of 38 species in Grp-noAB HPV+ group (63.2%) were recognized for their involvement in periodontal disease by performing manual searches in PubMed (Supplementary Table 3).

### Receiver-Operating Characteristic Determination

Using the Mann–Whitney *U*-test, 31 of 43 bacterial species in Grp-All HPV+ and 29 of 38 bacterial species in Grp-noAB HPV+ LEfSe were significant (*p* < 0.05) (Figures 3A,B). Using MedCalc ROC curve analysis, one species (*Lachnoanaerobaculum sp083*) in Grp-All HPV+ was found not significant. All species in Grp-noAB HPV+ were, however, significant (*p* < 0.01). By minimizing zero inflation, we found 17 of the 31 Grp-All HPV+ species and 16 of the 29 Grp-noAB HPV+ species to be significant (MedCalc Software Ltd, Ostend, Belgium). Minimization of zero inflation is required to optimize ROC analysis for the bacterial species which are more consistently detected across subjects and to increase the “signal-to-noise” ratio for a panel of select candidate bacterial taxa biomarkers. Indeed, *Haemophilus pittmaniae*, *Rumonococcaceae G1* sp. *HOT 075*, and three *Leptotrichia* spp. were determined to be “Excellent” biomarkers in terms of sensitivity, specificity, and accuracy in the Grp-noAB using the log(RA + 1) transformed data with minimized zero inflation (Supplementary Table 4). Descriptive statistics of all the species with significant ROC curves are presented in Supplementary Table 4. Notably, *Leptotrichia* was the most represented significant genus for both Grp-All and Grp-noAB groups HPV+ vs. HPV– comparisons.

### CombiROC and Microbial Interaction Network Database Investigation

**FIGURE 4:**
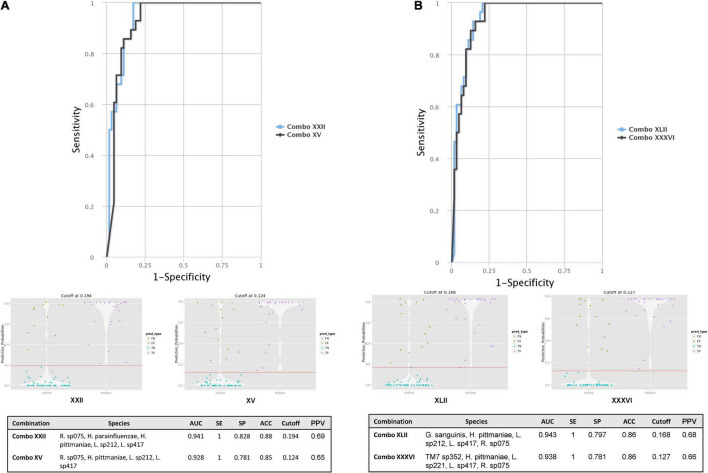
HNC HPV+ vs. HNC HPV– CombiROC analysis using RA and log(RA + 1) transformed abundance data. **(A)** CombiROC analysis for biomarkers with AUC > 0.75 on RA data. **(B)** CombiROC analysis for biomarkers with AUC > 0.75 on log(RA + 1) data. Significant (α = 0.05) CombiROC program-generated receiver operator characteristic (ROC) curves for **(A)** RA data and **(B)** log(RA + 1) transformed abundance data of candidate microbial biomarkers (AUC > 0.75; *n* = 10 bacterial species) identified *via* MedCalc Software for Grp-All HPV+ vs. HPV– group are shown. **(A)** ROC curves, violin plots, and descriptive statistic summary table for the best two combinations from 10 potential markers using the RA are shown. “Combo XXII” consists of a combination of the markers *Ruminococcaceae sp075*, *Haemophilus parainfluenzae*, *Haemophilis pittmaniae*, *Leptotrichia sp212*, and *Leptotrichia sp417* (ROC curve blue line). “Combo XV” contains markers *Ruminococcaceae sp075*, *H. pittmaniae*, *Leptotrichia sp212*, and *Leptotrichia sp417* (ROC curve black line). **(B)** ROC curves, violin plots, and descriptive statistic summary table for the best two combinations from 10 potential markers using the log(RA + 1) transformed data are shown. “Combo XLII” contains potential markers *Gemella sanguinis*, *H. pittmaniae*, *Leptotrichia sp212*, and *Ruminococcaceae sp075* (ROC curve blue line). “Combo XXXVI” consists of species probes *TM7 G1 sp352*, *H. pittmaniae*, *Leptotrichia sp221*, *Leptotrichia sp417*, and *Ruminococcaceae sp075* (ROC curve black line). Cut-off refers to the optimal value or the highest true positive rate that has the lowest false positive rate. FN, false negative; FP, false positive; TN, true negative; TP, true positive; AUC, area under the curve; SE, sensitivity; SP, specificity; ACC, accuracy; PPV, positive predictive value.

**FIGURE 5:**
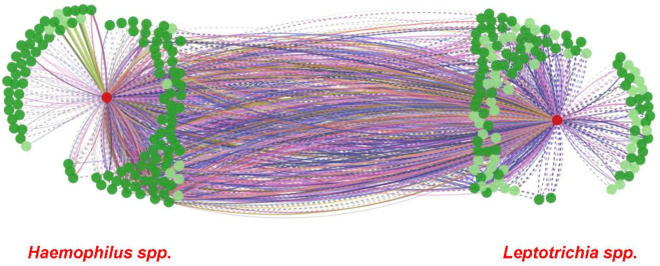
MIND interaction network of *Haemophilus* and *Leptotrichia* spp. Microbial Interaction Network Database (MIND; http://www.microbialnet.org/mind_home.html) results, illustrating a network between *Haemophilus* spp. (left red circle) and *Leptotrichia* spp. (right red circle) by using MIND default options, are presented. Dark green circles depict genera that interact with *Haemophilus* spp./*Leptotrichia* spp., and light green circles depict species that interact with *Haemophilus* spp./*Leptotrichia* spp. The lines between *Haemophilus* spp. and *Leptotrichia* spp. show interactions in common with other genera and species. The different color lines depict the human tissue sites where interactions have been demonstrated in previous studies (MIND; http://www.microbialnet.org/mind_home.html).

From the ROC MedCalc analyses, 10 bacterial species from Grp-All HPV+ group had an AUC of at least 0.75, distinguishing HNC HPV+ from HNC HPV– group (Supplementary Table 4). Using RA data in CombiROC program (Mazzara et al., 2017), 24 “gold” combinations were generated out of 2,036 possible combinations containing at least two markers. The best two combinations (greatest AUC) were “Combo XXII” consisting of the microbial species probes *Ruminococcaceae sp075*, *H. parainfluenzae*, *H. pittmaniae*, *Leptotrichia sp212*, and *Leptotrichia sp417* and “Combo XV” consisting of *Ruminococcaceae sp075*, *H. pittmaniae*, *Leptotrichia sp212*, and *Leptotrichia sp417* (Figure 4A). “Combo XXII” and “Combo XV” had AUCs of 0.941 and 0.928, accuracies of 0.88 and 0.85, and positive predictive values of 0.69 and 0.65, respectively (Figure 4A). Using log(RA + 1) data, 46 “gold” combinations were created out of 1,013 possible combinations with the best two combinations of microbial probes being “Combo XLII” and “Combo XXXVI.” “Combo XLII” contained a combination of *Gemella sanguinis*, *H. pittmaniae*, *Leptotrichia sp212*, and *Ruminococcaceae sp075*, while “Combo XXXVI” contained *TM7 G1 sp352*, *H. pittmaniae*, *Leptotrichia sp221*, *Leptotrichia sp417*, and *Ruminococcaceae sp075* (Figure 4B). “Combo XLII” and “Combo XXXVI” had AUCs of 0.943 and 0.938 and positive predictive values of 0.68 and 0.66, respectively. Both of these combinations from log(RA + 1) transformed data had an accuracy of 0.86 (Figure 4B). ROC curves, violin plots, and descriptive statistics of each data type are presented in Figure 4. Using MIND, *Haemophilus* and *Leptotrichia* were found to have many interactions in common (Figure 5).

### LitSuggest Performance

From the Python**_v3.6.2_** data extraction code, 203 PubMed articles were identified for classification from the model matching the search terms “HPV” and “microbiome.” From the classification set of these articles, 36 were determined as positively associated with the search terms “oral microbiome” and “HPV.” LitSuggest program found 171 articles negatively classified. Manual validation of the 36 positively associated articles resulted in 21 articles being discarded. A total of 15 remaining articles were correctly determined as positively associated with HPV and the oral microbiome. Of these articles, three were reviews and 12 were research articles evaluating the HPV status in the context of oral tumor and microbiome relationship in SCC patients with oropharynx, including tonsil specifically, as the primary tumor site (Table 3).

**TABLE 3:** LitSuggest positively classified articles (*n* = 15) involving HPV and the oral microbiome in HNC patients.

Year^a^	Author^b^	Purpose of study^c^	Findings^d^	PMID^e^
2021	Gougousis et al., 2021 (review)	Review the significance of biomarkers based on epigenetics and microbiome profile in the diagnosis of HPV-related OSCC.	*Streptococcus salivarius* (+), *Streptococcus gordonii* (+), *Gemella haemolysans*, *Gemella morbillorum* (+), *Johnsonella ignava* (+), and *Streptococcus parasanguinis* (+) highly associated with tumor site. *Gemella adiacens* (+) association with non-tumor site. HPV+ correlation between the genera *Haemophilus* and *Gemella* in oral cavity cancer. *Actinomyces* (+), *Parvimonas* (+), *Selenomonas* (+), and *Prevotella* (+) in OCC compared to OPC. *Corynebacterium* (+) and *Kingella* (+) are associated with decreased risk of oral cancer.	33521000
2021	De Keukeleire et al., 2021 (review)	Knowledge and biomarkers in HNC-SCC.	HPV is a biomarker of HNC-SCC; *Lactobacilli* (+); *Haemophilus* (–); *Neisseria* (–); *Gemellaceae* (–); *Aggregatibacter* (–); *Streptococci* (–); *Fusobacteria* (+); *Fusobacterium nucleatum* (+) associated with lower tumor stage	33916646
2021	De Martin et al., 2021	Characterize microbiome of human palatine tonsil crypts in patients with high-risk HPV-associated tonsil cancer compared to sleep apnea controls.	*Firmicutes* (+); *Actinobacteria* (+); *Veillonella* (+); *Streptococcus* (+); *Prevotella* (+); *Filifactor alocis* and *Prevotella melaninogenica* were distinct features of tonsil cancer	34367729
2021	Oliva et al., 2021	Characterize oral and gut microbiome of HPV+ OSCC patients before and after CRT.	*F. nucleatum* (+), *G. morbillorum* (+), *G. haemolysans* (+), *Leptotrichia hofstadii* (+), *Selenomonas sputigena* (+), and *Selenomonas infelix* (+) in stage III OSCC	33750907
2021	Rajasekaran et al., 2021	Characterize microbiome in patients with HPV-associated early tonsil SCC compared to benign tonsil specimens.	*Burkholderia pseudomallei* was unique to cancer specimens. *Fusobacteria* was identified in HPV-associated OSCC patients in tonsil and lymph node specimens. Negative nodes showed signatures for *Anaplasma phagocytophilum*, *Bacillus subtilis*, *Chlamydia trachomatis*, *Chlamydophila psittaci*, *Lactococcus lactis*, and *Proteus mirabilis*	33905914
2021	Shigeishi et al., 2021a	Characterize HPV16 DNA prevalence and PD inflammation in older Japanese women.	*Prevotella intermedia* (+), *Porphyromonas* (+), *Veillonella* (–), and *Prevotella* (–) in HPV+ periodontal inflammation	33456534
2021	Shigeishi et al., 2021b (review)	Review recent findings of oral HPV infection in relation to periodontitis.	HPV localizes to inflammatory periodontal tissue, and periodontal pockets may act as a reservoir for HPV. Smoking is associated with HPV and periodontitis. Carcinogenic HPV and periodontitis may lead to OCC, but HPV E6/E7 has not been fully investigated in patients with periodontitis. Oral HPV prevalence may be associated with periodontitis.	33728046
2021	Zakrzewski et al., 2021	Compare microbial composition, diversity, and specific bacterial phytotypes between HPV+ and HPV– oropharyngeal tumors using saliva, normal tissue, and tumor tissue.	*Treponema* (+) and *Spirochaetes* (+) were associated with normal tissues of HPV+ patients; *Neisseria*, *Veillonella*, *Fusobacterium*, *P. melaninogenica*, and *Porphyromonas* were associated with HPV status (not significant). *Fusobacteria* (–) in saliva samples (not significant); *Leptotrichia* (–) in HPV-; *Rothia* (–) in HPV+ tumor tissues; *Atopobium* (–) in normal tissue HPV+ patients.	34278648
2020	Bahig et al., 2020	Characterize tumor microenvironment of HPV-associated OSCC with RT +/– cisplatin-based chemotherapy using surface swab of tonsil, base of tongue, and buccal mucosa.	Decreased α-diversity over course of treatment. *Veillonella* (+) and *Leptotrichia* (+) at tumor site. *Actinomyces* (–) and *Leptotrichia* (–) over the course of radiation. *Gemella* (–) and *Streptococcus* (–) between baseline and 1 week and returned to baseline at week 5. *Veillonella* (+) and *Topobium* (+) at week 5.	33367119
2019	Chowdhry et al., 2019	Explore deep-seated infected granulation tissue removed during periodontal flap surgery procedures for residential bacterial species between HPV+ and HPV– chronic periodontitis patients.	Deep-seated granulation tissues showed *Firmicutes* (+), *Proteobacteria* (+), and *Bacteroidetes* (+). *Veillonella arula* (+), *Selenomonas noxia* (+), *Neisseria oralis* (+), *P. intermedia* (+), *Prevotella nigrescens* (+), *Capnocytophaga ochracea* (+) in HPV+ samples. *Prevotella* (+), *Macellibacteroides fermentans* (+), *Porphyromonas endodontalis* (+), *Campylobacter rectus* (+), *Treponema phagedenis* (+) in HPV– samples. *Pseudoxanthomas kaohsiungensis* (+) in females and *Desulfobulbus rhabdoformis* (+) in males.	31111067
2018	Lim et al., 2018	Characterize the oral microbiome fluctuation associated with OCC and OSCC compared to healthy controls using oral wash samples.	*Rothia* (–), *Haemophilus* (–), *Corynebacterium* (–), *Paludibacter* (–), *Porphyromonas* (–), *Capnocytophaga* (–) in OCC and OSCC. *Oribacterium* (+) in OCC and OSCC. *Actinomyces* (+), *Parvimonas* (+), *Selenomonas* (+), and *Prevotella* (+) in OCC compared to OSCC. *Haemophilus* (+), *Gemella* (+) with HPV+. *Actinomyces* (+), *Actinobacillus* (+), *Lautropia* (+), *Fusobacterium* (+), *Aggregatibacter* (+) in high-risk individuals. Panel of bacterial species *Rothia*, *Haemophilus*, *Corynebacterium*, *Paludibacter*, *Porphyromonas*, *Oribacterium*, and *Capynocytophaga* showed an area under curve of 0.98, sensitivity of 100%, and specificity of 90%	30123780
2018	Tuominen et al., 2018	Investigate the association between HPV infection and microbiome composition in the placenta, uterine cervix, and mouth in women	*Selenomonas* (+), *TM7* (+), *Megasphaera* (+) with HPV+ in oral samples. *Haemophilus* (+) with HPV– oral samples. Higher richness in HPV+ than HPV– samples.	29955075
2017	Guerrero-Preston et al., 2017	Characterize microbial species in the saliva microbiome and tumor characteristics in HNC-SCC patients.	*Veillonella dispar* (+) in all samples. *S. salivarius* (+), *Streptococcus vestibularis* (+) in HNC-SCC samples. *Lactobacillus* spp. (+), *Parvimonas micra* (+), *Streptococcus mutans* (+), and *F. nucleatum* (+) in salivary HNC-SCC samples. *Fusobacterium periodonticum* (–), *Leptotrichia trevisanii* (–), *L. hofstadii* (–), and *Leptotrichia* (–) in HNC-SCC compared to controls. Lower diversity in HNC-SCC than controls regardless of HPV status. No significant differences when comparing HPV+ to HPV– saliva HNC-SCC samples with control. *F. periodonticum* (+) in saliva from HNC-SCC patients. *Lactobacillus rhamnosus* (+), *Lactobacillus salivarius* (+), *Lactobacillus vaginalis* (+), *Lactobacillus reuteri* (+), *Lactobacillus fermentum* (+), *Lactobacillus johnsonii* (+), *Lactobacillus gasseri* (+) in subset of HNC-SCC samples from Johns Hopkins University. *Lactobacillus* was 710 time higher, and *L. vaginalis* was 52 times higher in HNC-SCC samples compared to controls.	29340028
2017	Wolf et al., 2017	Compare oral salivary microbiome samples of patients with OCC and OSCC vs. healthy controls.	Shannon index found higher diversity in tumor patients but was not significant. Highest LEfSe LDA was from *Proteobacteria*. *Prevotella* (+), *Haemophilus* (+), *Neisseria* (+), *Streptococcus* (+), and *Veillonella* (+) in healthy controls. *Actinomyces* (+), *Schwartzia* (+), *Treponema* (+), and *Selenomonas* (+) in HNC-SCC patients. HPV+ patients demonstrated normal microbiome compared to healthy controls.	28725009
2016	Guerrero-Preston et al., 2016	Compare saliva microbiome from HPV+ and HPV–, OCC, OSCC, and normal cavity epithelium.	*Firmicutes* (+), *Proteobacteria* (+), *Bacteroidetes* (+), *Actinobacteria* (–), and Fusobacteria (–) prior to surgery. At lower levels *Streptococcus* (+), *Prevotella* (+), *Haemophilus* (+), *Lactobacillus* (+), *Veillonella* (+), *Citrobacter* (–), *Kingella* (–) prior to surgery. HNC-SCC patients exhibited lower richness and diversity compared to controls. *Streptococcus*, *Dialister*, and *Veillonella* were able to discriminate tumor from control samples. *Neisseria* (–), *Aggregatibacter* (–), *Haemophilus* (–), and *Leptotrichia* (–) in tumor samples. *Enterobacteriaceae* and *Oribacterium* discriminate OCC from OSCC and normal samples. *Gemellaceae* (+) and *Leuconostoc* (+) only observed in HPV+ samples. α-diversity was reduced post-surgery.	27259999

*Using Python_v3.6.2_ program, 203 PubMed articles were retrieved for classification from the model that matched the search terms “HPV” and “microbiome.” LitSuggest program determined 36 articles to be positively associated with “HPV” and “oral microbiome.” Manual validation of the 36 positively classified articles resulted in 21 articles being discarded. The remaining 15 articles were manually validated as positively classified articles that relate to HNC, HPV, and the oral microbiome.*

*^a^Year of publication.*

*^b^First listed author.*

*^c^Purpose or outcomes explored during the study.*

*^d^Findings/results of the study.*

*^e^PubMed ID. HPV, human papillomavirus; HNC, head and neck cancer; OSCC, oral squamous cell carcinoma; OCC, oral cavity cancer; OPC, oropharyngeal cancer; SCC, squamous cell carcinoma; CRT, chemoradiotherapy; PD, Parkinson’s disease; LDA, linear discriminant analysis; RT, radiotherapy.*

## Discussion

This is the first study to evaluate the microbial differences in HNC HPV+ patients compared to those of healthy individuals and HNC HPV– patients by means of oral samples including saliva, buccal mucosa, supragingival plaque, and tongue swabs using multivariate analysis. We were able to identify 442 species and 65 genera detected based on HOMI*NGS* sequencing data. We confirmed findings from multiple studies indicating that shifts in microbiome profiles which may be defined as “dysbiosis” occur in HNC patients compared to HC subjects (Guerrero-Preston et al., 2016, 2017; Tuominen et al., 2018).

Furthermore, we were able to establish that HNC HPV+ patients have significantly different microbiome than that of HNC HPV– patients (Supplementary Table 2). While α-diversity was not significantly different between HNC HPV+ and HNC HPV– patients, α-diversity differed between HNC patients and HC subjects (Table 2). Additionally, β-diversity differences were significant for all comparisons in this study except for one out of 30 comparisons (Supplementary Table 2). There was a clear separation between the Grp-All HNC patients and HC subjects (Supplementary Figure 1A). We were also able to determine that although antibiotic treatment within 2 weeks of sampling is a confounding variable, excluding antibiotic-treated HNC patients did not affect the main results, by comparing the microbiome data of HNC HPV+ patients with the data of HNC HPV– patients (i.e., GrpAll and GrpNoAB) (Supplementary Table 1). A visualization of the division of the Grp-All HNC HPV+ vs. HNC HPV– can be seen in Supplementary Figure 1B.

Regarding periodontal disease and dental caries status, our sub-cohort is similar to that of the larger OraRad cohort (Table 1 and Brennan et al., 2021). A study identified by LitSuggest, reviewing findings pertaining to oral HPV infection in relation to periodontitis, suggests periodontal pockets may act as a reservoir for HPV and that oral HPV prevalence may be associated with periodontitis (Shigeishi et al., 2021a). Another recent study characterizing HPV16 DNA prevalence and periodontal disease inflammation in a population of older Japanese women identified an increase of *Prevotella intermedia* and *Porphyromonas* and a decrease of *Veillonella* and *Prevotella* to be associated with periodontal disease inflammation (Shigeishi et al., 2021b).

Furthermore, a study by Chowdhry et al. (2019) exploring deep-seated infected tissues removed during periodontal flap surgery in chronic periodontitis patients, observed an increased abundance of *Veillonella arula*, *Selenomonas noxia*, *Neisseria oralis*, *P. intermedia*, *Prevotella nigrescens*, and *Capnocytophaga ochracea* in HPV+ samples. Interestingly, in our study we found species of *Capnocytophaga*, *Neisseria*, *Prevotella*, *Selenomonas*, and *Veillonella* spp. to represent distinct taxa for HNC HPV+ patients through LEfSe analysis (Figure 3). Species from these genera were also found to be significant using Mann–Whitney *U*-test (*p* < 0.05) (Figure 3). While ROC curves were also significant (*p* < 0.01), we determined none of these species to be excellent biomarkers (Supplementary Table 4). We agree with Shigeishi et al. (2021b) suggesting that sampling methods of the oral microbiome should be carefully selected for periodontal tissue to ensure detection of HPV DNA directly along with the associated periodontal microbiome.

LEfSe analysis showed 43 bacterial species differentiating Grp-All HNC HPV+ from Grp-All HNC HPV– patients (Figure 3). Grp-noAB group analysis confirmed these findings since 24 bacterial species characterizing the HNC HPV+ patients were in common with the species distinctive of Grp-All HNC HPV+ patients (Figure 3). *Leptotrichia* spp. were the most prominent and significant species in comparisons performed for both Grp-All and Grp-noAB groups, precluding the possibility that antibiotics alone account for differences between HNC HPV+ and HPV– groups. In addition to LEfSe, Mann–Whitney *U-*tests comparing the RA of HNC HPV+ to HNC HPV– found 5/6 (83%) and 6/6 (100%) *Leptotrichia* spp. for Grp-All and Grp-noAB, respectively, to be significant (*p* < 0.05) (Figure 3). For these *Leptotrichia* spp., the RA was found to be greater in samples of HNC HPV+ compared to HNC HPV– patients for Grp-All and Grp-noAB (data not shown). ROC analysis on log(RA + 1) data further confirmed these findings with five Grp-All *Leptotrichia* spp. and six Grp-noAB *Leptotrichia* spp. to have an AUC significantly different from that of 0.5 (*p* < 0.01) (Supplementary Table 4). By minimizing zero inflation on log(RA + 1) transformed abundance data for the Grp-noAB group, we determined three *Leptotrichia* spp. (*Leptotrichia sp215*, *sp392*, and *sp417*) to be excellent biomarkers distinguishing HNC HPV+ from HNC HPV– patients, with a sensitivity >95%, a specificity = 100%, and an accuracy >95% (Supplementary Table 4D).

A previous study by Bahig et al. (2020) investigating the tumor microenvironment of HPV-associated SCC patients, determined an increased abundance of *Leptotrichia* genus in oral samples at baseline which declined over the course of radiation. This study was positively classified in our LitSuggest analysis (Table 3). A study by Oliva et al. (2021) found that *Leptotrichia hofstadii* was abundant in stage III oropharynx cancer, while Zakrzewski et al. (2021) determined *Leptotrichia* genus to be decreased in oropharynx HPV- tumor samples.

Surprisingly, other studies have found *Leptotrichia* spp. to be absent or less abundant at SCC primary tumor sites (Schmidt et al., 2014; Guerrero-Preston et al., 2016, 2017). *Leptotrichia* spp. have also been investigated for their role in periodontal disease (i.e., gingivitis and periodontitis) (Supplementary Table 3; Eribe and Olsen, 2017). A systematic review by Pérez-Chaparro et al. (2014) described a study by Griffen et al. (2012) that correlated an increased abundance of *Leptotrichia* genera, *Leptotrichia oral taxon 210*, *Leptotrichia EX103*, and *Leptotrichia IK040* to be associated with deep pockets of patients with periodontal disease. Accordingly, *Leptotrichia* species consist of non-motile facultative anaerobic and anaerobic species mostly present in the oral cavity (Eribe and Olsen, 2017).

We also observed that *H. pittmaniae* had a higher RA in HNC HPV+ than HNC HPV– patients in Grp-All and Grp-noAB (data not shown). This species was identified as a differential feature of HPV+ by LEfSe and was found to have a significant ROC curve using log(RA + 1) data with an AUC of 0.824 (Figure 3 and Supplementary Table 4). Additionally, this species was determined to be an excellent biomarker in the Grp-All and Grp-noAB log(RA + 1) ROC curve analysis with zero inflation minimized as well as a good biomarker when zero inflation was not minimized (Supplementary Table 3). *H. pittmaniae* was also included in all four multi-marker ROC combinations including *Leptotrichia* species, suggesting it is a contributor to HPV+ SCC progression (Figure 4). *H. pittmaniae* has been suggested as a pathogen possibly responsible for respiratory tract infections in patients with lung diseases (Boucher et al., 2012) but has also been identified at significantly higher levels in male children with active caries (Ortiz et al., 2019). While little is known about *H. pittmaniae* and its role in periodontal disease, the *Haemophilus* genus was identified in many positively classified studies per our LitSuggest text mining analysis. In a recent study by De Keukeleire et al. (2021) a decrease in *Haemophilus* was associated with HNC-SCC, confirming findings by Wolf et al. (2017) and Lim et al. (2018). However, studies by Guerrero-Preston et al. (2016) and Gougousis et al. (2021) found the opposite to be true. In our study, we were able to verify findings by Lim et al. (2018) and Tuominen et al. (2018) that an increase in abundance of *Haemophilus* in the oral cavity is associated with HNC-SCC HPV+ samples. Altogether, a microbiome metagenome/metatranscriptome survey focused on *Haemophilus* and *Leptotrichia* at the species and strain levels could provide reliable biomarker signatures with clinical implications for HNC HPV+ patients in the future.

MIND analysis found many microbial interactions between the genera *Leptotrichia* and *Haemophilus* connected through many species and genera (Figure 5). These genera have also been shown as two of the nine taxa that facilitate structures in oral plaque and intermingle at the micron scale (Mark Welch et al., 2016). The study by Mark Welch et al. (2016) also suggests that *Corynebacterium* forms long structures with *Streptococcus* and *Porphyromonas* in direct contact. *Streptococcus* creates an environment rich in CO_2_, lactate, and acetate, facilitating the contact with *Haemophilus*, *Aggregatibacter*, and *Neisseriaceae* (Mark Welch et al., 2016). With the exception of *Neisseriaceae*, these species are likely essential to aerobic metabolism, allowing *Fusobacterium* and *Leptotrichia* to thrive as key participants in the metabolism of sugars, producing lactic acid (Mark Welch et al., 2016). It is suggested that this metabolism is involved in degradation of oral tissues, possibly leading to dental caries and/or periodontal disease (Eribe and Olsen, 2017).

Text mining used in this study suggests a lack of information involving HPV and the oral microbiome of HNC patients. Of 203 HPV microbiome articles, only 15 were verified as relevant to HPV and the oral microbiome in SCC. Only two studies identified by LitSuggest related oral SCC with HPV and periodontitis (Chowdhry et al., 2019; Shigeishi et al., 2021a). Furthermore, few studies investigated multiple primary tumor sites in SCC patients. Most identified studies in our analysis focused on investigating oropharyngeal SCC (Guerrero-Preston et al., 2016; Wolf et al., 2017; Lim et al., 2018; Bahig et al., 2020; Gougousis et al., 2021; Oliva et al., 2021; Zakrzewski et al., 2021). Only two positively classified articles were found to characterize the microbiome in the tonsil of SCC patients (Table 3; De Martin et al., 2021; Rajasekaran et al., 2021). In the future, more studies on HNC-SCC HPV+ patients using larger patient cohorts will be required to determine HNC risk in relation to the oral microbiome and HPV status.

### Limitations

While this study was able to show the significance of microbial composition in HNC-SCC HPV+ patients compared to HNC-SCC HPV– patients, we were unable to account for the immune status of the patients. Furthermore, our patient cohort was relatively small, and our design was not optimally balanced due to the various primary cancer sites in our patient cohort. In addition, many factors not addressed in this study may affect HNC progression, such as genetics, oral hygiene practices, and periodontal treatment. However, our main conclusion remains pertinent, in that the species identified as multi-marker combinations, i.e., *H. pittmaniae* and *Leptotrichia* spp., increase in HNC-SCC HPV+ patients regardless of the primary cancer site.

## Data Availability Statement

The data presented in the study are provided as Supplementary Material and have been deposited in a public GitHub repository. The data can be found here: https://github.com/mbeckm01/HPV_HNC.git.

## Ethics Statement

The studies involving human participants were reviewed and approved by the Atrium Health Institutional Review Board and University of Connecticut Health Institutional Review Board. The patients/participants provided their written informed consent to participate in this study.

## Author Contributions

J-LM and FB conceived this microbiome study. MTB and RL had previously established the cited clinical outcomes study “OraRad” and provided clinical insights for this study. J-LM directed the statistical analyses implemented and verified by MFB and HL. MFB, HL, J-LM, and FB contributed to the writing of the manuscript, the overall analysis, and biological interpretation. All authors participated in the revisions of the manuscript and interpretation of the results, gave their final approval, and agreed to be accountable for all aspects of the work.

## Conflict of Interest

The authors declare that the research was conducted in the absence of any commercial or financial relationships that could be construed as a potential conflict of interest.

## Publisher’s Note

All claims expressed in this article are solely those of the authors and do not necessarily represent those of their affiliated organizations, or those of the publisher, the editors and the reviewers. Any product that may be evaluated in this article, or claim that may be made by its manufacturer, is not guaranteed or endorsed by the publisher.

## Footnotes

1
https://www.cancer.gov/about-cancer/causes-prevention/risk/infectious-agents/hpv-and-cancer

2
http://combiroc.eu

3
https://www.ncbi.nlm.nih.gov/research/litsuggest

4
https://github.com/mbeckm01/HPV_HNC.git
