# Supplementary material for: Haemophilus pittmaniae and Leptotrichia spp. Constitute a Multi-Marker Signature in a Cohort of Human Papillomavirus-Positive Head and Neck Cancer Patients
Source: Front Microbiol. 2022 Jan 18;12:794546. doi: 10.3389/fmicb.2021.794546 (PMC8803733; doi:10.3389/fmicb.2021.794546)
Supplement: Supplementary file 1 [file Table_1.docx]

**Supplemental Table 1. Species and genera detected by Human Oral Microbiome Identification, HOMI*NGS***

| **Comparison^a^** | **Avg. species detected^b^** | **p-value^c^** | **Avg. genera detected^d^** | **p-value^e^** |
| --- | --- | --- | --- | --- |
| **HNC** | 105.38 | 0.008 | 25.38 | 0.507 |
| **HC** | 116.46 |  | 25.84 |  |
| **HNC Grp-noAB** | 108.28 | 0.049 | 25.17 | 0.528 |
| **HC Grp-noAB** | 116.46 |  | 25.84 |  |
| **HNC BPST** | 106.16 | 0.025 | 25.49 | 0.611 |
| **HC BPST** | 117.66 |  | 25.97 |  |
| **HNC BPST Grp-noAB** | 105.74 | 0.033 | 25.43 | 0.806 |
| **HC BPST Grp-noAB** | 117.66 |  | 25.84 |  |
| **HNC HPV+** | 110.34 | 0.040 | 25.68 | 0.868 |
| **HNC HPV-** | 96.08 |  | 25.33 |  |
| **HNC HPV+ Grp-noAB** | 105.42 | 0.760 | 24.75 | 0.341 |
| **HNC HPV- Grp-noAB** | 102.00 |  | 26.83 |  |

Footnote:

**^a^**Comparisons of the average number of species and genera detected per subject included head and neck cancer (HNC) *vs.* healthy controls (HC), HNC-HPV-positive (HPV+) *vs.* HNC-HPV-negative (HPV-), with/ without antibiotic (AB) treatment within two-weeks of sampling, for any sample site yielding microbiome data, *i.e.*, B (buccal), P (plaque), S (saliva), and/ or T (tongue). Comparisons for subjects who had microbiome data for all four sample sites combined (BPST) are also shown.

**^b^**The average number of species detected for each comparison.

**^c^**p-value for each comparison of species detected using Mann-Whitney U-test.

**^d^**The average number of genera detected for each comparison.

**^e^**p-value for each comparison of genera detected using Mann-Whitney U-test.
