# Supplementary material for: Haemophilus pittmaniae and Leptotrichia spp. Constitute a Multi-Marker Signature in a Cohort of Human Papillomavirus-Positive Head and Neck Cancer Patients
Source: Front Microbiol. 2022 Jan 18;12:794546. doi: 10.3389/fmicb.2021.794546 (PMC8803733; doi:10.3389/fmicb.2021.794546)
Supplement: Supplementary file 2 [file Table_2.docx]

|  | **Monte-Carlo p-value** | | | **Monte-Carlo p-value** | |
| --- | --- | --- | --- | --- | --- |
| **Comparisons^a^** | **Grp-All** | | | **Grp-NoAB** | |
| **HNC vs. HC** | **Diagnosis** | **Sample Site** | **Antibiotics** | **Diagnosis** | **Sample Site** |
| BPST | 0.0001 | 0.0001 | 0.0009 | 0.0001 | 0.0001 |
| BST | 0.0226 | 0.0001 | 0.0002 | 0.0004 | 0.0001 |
| PST | 0.0009 | 0.0001 | 0.0168 | 0.0001 | 0.0001 |
| **Comparisons^b^** | **Grp-All** | | | **Grp-NoAB** | |
| **HPV vs. HC** | **HPV Status** | **Sample Site** | **Antibiotics** | **HPV Status** | **Sample Site** |
| BPST | 0.0001 | 0.0001 | 0.0113 | 0.0001 | 0.0001 |
| BST | 0.0019 | 0.0001 | 0.0008 | 0.0001 | 0.0001 |
| PST | 0.0001 | 0.0001 | 0.0667 | 0.0001 | 0.0001 |
| **HPV+ vs. HC** | **HPV Status** | **Sample Site** | **Antibiotics** | **HPV Status** | **Sample Site** |
| BPST | 0.0216 | 0.0001 | 0.0012 | 0.0028 | 0.0001 |
| BST | 0.261 | 0.0027 | 0.0009 | 0.0016 | 0.0001 |
| PST | 0.0393 | 0.0002 | 0.01 | 0.002 | 0.0001 |
| **HPV- vs. HC** | **HPV Status** | **Sample Site** | **Antibiotics** | **HPV Status** | **Sample Site** |
| BPST | 0.0001 | 0.0001 | 0.4237 | 0.0001 | 0.0001 |
| BST | 0.0039 | 0.0005 | 0.0646 | 0.0004 | 0.0001 |
| PST | 0.0004 | 0.0001 | 0.4903 | 0.0001 | 0.0001 |
| **HPV+ vs. HPV-** | **HPV Status** | **Sample Site** | **Antibiotics** | **HPV Status** | **Sample Site** |
| BPST | 0.0001 | 0.0001 | 0.1262 | 0.0001 | 0.0001 |
| BST | 0.0024 | 0.0001 | 0.0067 | 0.0001 | 0.0001 |
| PST | 0.0024 | 0.0001 | 0.3426 | 0.0184 | 0.0006 |

**Supplemental Table 2. *Beta*-diversity PERMANOVA analyses**

Footnote:

PERMANOVA Monte-Carlo corrected p-values for Grp-All and Grp-noAB (no antibiotic treatment) comparisons are shown.

**^a^**Comparisons with analytical design of ‘Diagnosis’ and ‘Sample site’ as fixed variables and ‘Antibiotics’ random and nested into ‘Diagnosis’ and ‘Sample site’. This analysis was completed for sample site combinations BPST, BST, and PST for comparison HNC *vs.* HC.

**^b^**Comparisons with analytical design of ‘HPV Status’ and ‘Sample site’ as fixed variables and ‘Antibiotics’ random and nested into ‘Diagnosis’ and ‘Sample Site’. This analysis was completed for sample site combinations BPST, BST, and PST for comparisons of HPV *vs.* HC, HPV+ *vs.* HC, HPV- *vs.* HC, and HPV+ *vs.* HPV-.
