# Supplementary material for: Haemophilus pittmaniae and Leptotrichia spp. Constitute a Multi-Marker Signature in a Cohort of Human Papillomavirus-Positive Head and Neck Cancer Patients
Source: Front Microbiol. 2022 Jan 18;12:794546. doi: 10.3389/fmicb.2021.794546 (PMC8803733; doi:10.3389/fmicb.2021.794546)
Supplement: Supplementary file 3 [file Table_3.docx]

| **LEfSe Grp-All HPV + Species** | **PMID** |
| --- | --- |
| *Actinomyces graevenitzii* | ND |
| *Actinomyces israelii* | 26324012 |
| *Actinomyces sp HOT 877* | 25074492 |
| *Alloprevotella tannerae* | 25074492 |
| *Butyrivibrio sp HOT 455* | 23673380 |
| *Campylobacter curvus* | ND |
| *Capnocytophaga sp HOT 326* | 31384554 |
| *Capnocytophaga sp HOT 332* | 31384554 |
| *Capnocytophaga sputigena* | 31384554 |
| *Cardiobacterium hominis* | ND |
| *Catonella morbi* | 28748030 |
| *Fusobacterium periodonticum* | 16464693 |
| *Gemella sanguinis* | ND |
| *GN02 G1 sp HOT 872* | ND |
| *Haemophilus parainfluenzae* | ND |
| *Haemophilus pittmaniae* | ND |
| *Johnsonella sp HOT 166* | 25074492 |
| *Lachnoanaerobaculum orale* | 31681625 |
| *Lachnoanaerobaculum sp HOT 083* | 31681625 |
| *Lachnospiraceae G2 sp HOT 096* | 31681625 |
| *Leptotrichia sp HOT 212* | 25074492 |
| *Leptotrichia sp HOT 218* | 25074492 |
| *Leptotrichia sp HOT 221* | 25074492 |
| *Leptotrichia sp HOT 223* | 25074492 |
| *Leptotrichia sp HOT 417* | 25074492 |
| *Leptotrichia sp HOT 498* | 25074492 |
| *Megasphaera micronuciformis* | ND |
| *Neisseria elongata* | ND |
| *Neisseria oralis* | ND |
| *Oribacterium parvum* | ND |
| *Oribacterium sinus* | ND |
| *Porphyromonas pasteri* | ND |
| *Prevotella histicola* | ND |
| *Prevotella pleuritidis* | ND |
| *Ruminococcaceae G1 sp HOT 075* | ND |
| *Selenomonas sp HOT 136* | 25074492 |
| *SR1 G1 sp HOT 874* | 25074492 |
| *SR1 G1 sp HOT 875* | 25074492 |
| *Stomatobaculum sp HOT 097* | 25074492 |
| *Tannerella sp HOT 286* | 30581850 |
| *TM7 G1 sp HOT 352* | 25074492 |
| *TM7 G3 sp HOT 351* | 25074492 |
| *Veillonella rogosae* | ND |

**Supplemental Table 3. LEfSe HPV+ differential species involvement in periodontal disease**

**b.**

| **LEfSe Grp-noAB HPV+ Species** | **PMID** |
| --- | --- |
| *Actinomyces israelii* | 26324012 |
| *Actinomyces sp HOT 172* | 26324012 |
| *Aggregatibacter sp HOT 513* | ND |
| *Alloprevotella sp HOT 473* | ND |
| *Alloprevotella tannerae* | 25074492 |
| *Capnocytophaga sputigena* | 31384554 |
| *Catonella morbi* | 28748030 |
| *Eikenella corrodens* | 1474466 |
| *Fusobacterium periodonticum* | 16464693 |
| *Gemella sanguinis* | ND |
| *Haemophilus parainfluenzae* | ND |
| *Haemophilus pittmaniae* | ND |
| *Lachnoanaerobaculum orale* | 31681625 |
| *Lachnoanaerobaculum sp HOT 083* | 31681625 |
| *Lachnospiraceae G3 sp HOT 100* | 31681625 |
| *Leptotrichia sp HOT 212* | 25074492 |
| *Leptotrichia sp HOT 215* | 25074492 |
| *Leptotrichia sp HOT 221* | 25074492 |
| *Leptotrichia sp HOT 392* | 25074492 |
| *Leptotrichia sp HOT 417* | 25074492 |
| *Leptotrichia sp HOT 879* | 25074492 |
| *Megasphaera micronuciformis* | ND |
| *Neisseria oralis* | ND |
| *Olsenella sp HOT 809* | ND |
| *Oribacterium parvum* | ND |
| *Oribacterium sinus* | ND |
| *Peptococcus sp HOT 167* | 3074469 |
| *Prevotella histicola* | ND |
| *Prevotella nanceiensis* | ND |
| *Prevotella pallens* | ND |
| *Ruminococcaceae G1 sp HOT 075* | ND |
| *Selenomonas sp HOT 136* | 25074492 |
| *Selenomonas sp HOT 137* | 25074492 |
| *Selenomonas sputigena* | 25074492 |
| *SR1 G1 sp HOT 875* | 25074492 |
| *TM7 G1 sp HOT 352* | 25074492 |
| *TM7 G3 sp HOT 351* | 25074492 |
| *Veillonella rogosae* | 31904448 |

Footnote:

Periodontal disease involvement of LEfSe identified bacterial species of human papilloma virus positive (HPV+) patients for (**a**) Grp-All (n=43 bacterial species) and (**b**) Grp-noAB (n=38 bacterial species) using conventional search methods. PMID is PubMed ID; ND is not determined. Periodontal disease is understood as an infection with gingivitis representing an early form of the disease and periodontitis as an advanced form which may involve alveolar bone loss.
