# Supplementary material for: Haemophilus pittmaniae and Leptotrichia spp. Constitute a Multi-Marker Signature in a Cohort of Human Papillomavirus-Positive Head and Neck Cancer Patients
Source: Front Microbiol. 2022 Jan 18;12:794546. doi: 10.3389/fmicb.2021.794546 (PMC8803733; doi:10.3389/fmicb.2021.794546)
Supplement: Supplementary file 4 [file Table_4.docx]

**Supplemental Table 4. MedCalc ROC curve analysis results distinguishing HNC HPV+ group from HNC HPV- group**

| **Grp-All Species^a^** | **# HPV+^b^** | **# HPV-^c^** | **p-value^d^** | **AUC^e^** | **95% CI^f^** | **Sensitivity^g^** | **Specificity^h^** | **Associated Criterion^i^** | **Accuracy^j^** | **LR+^k^** | **LR-^l^** | **Quality^m^** |
| --- | --- | --- | --- | --- | --- | --- | --- | --- | --- | --- | --- | --- |
| ***Alloprevotella tannerae*** | 64 | 28 | 0.002 | 0.671 | 0.57-0.77 | 60.9 | 75 | 6.03x10-6 | 0.652 | 2.44 | 0.52 | Poor |
| ***Campylobacter curvus*** | 64 | 28 | 0.001 | 0.693 | 0.59-0.79 | 87.5 | 50 | 2.22x10-5 | 0.25 | 1.75 | 0.25 | Poor |
| ***Capnocytophaga sputigena*** | 64 | 28 | 0.001 | 0.678 | 0.57-0.77 | 46.9 | 85.7 | 3.78x10-5 | 0.576 | 3.28 | 0.62 | Poor |
| ***Cardiobacterium hominis*** | 64 | 28 | 0.001 | 0.658 | 0.55-0.75 | 50 | 82.1 | 6.03x10-6 | 0.598 | 2.8 | 0.61 | Poor |
| ***Catonella morbi*** | 64 | 28 | <0.001 | 0.703 | 0.59-0.79 | 57.8 | 82.1 | 1.59x10-5 | 0.641 | 3.24 | 0.51 | Poor |
| ***Fusobacterium periodonticum*** | 64 | 28 | <0.001 | 0.85 | 0.76-0.92 | 67.2 | 9.9 | 6.34x10-4 | 0.739 | 9.41 | 0.35 | Good/Poor |
| ***Gemella sanguinis*** | 64 | 28 | <0.001 | 0.766 | 0.67-0.85 | 85.9 | 60.7 | 5.35x10-5 | 0.772 | 2.19 | 0.23 | Good/Poor |
| ***Haemophilus parainfluenzae*** | 64 | 28 | <0.001 | 0.777 | 0.68-0.86 | 67.2 | 78.6 | 3.51x10-2 | 0.38 | 3.14 | 0.42 | Good/Poor |
| ***Haemophilus pittmaniae*** | 64 | 28 | <0.001 | 0.824 | 0.73-0.89 | 70.3 | 92.9 | 1.81x10-5 | 0.76 | 9.84 | 0.32 | Good |
| ***Lachnoanaerobaculum orale*** | 64 | 28 | <0.001 | 0.72 | 0.62-0.81 | 75 | 67.9 | 2.38x10-5 | 0.728 | 2.33 | 0.37 | Poor |
| ***Lachnospiraceae G2 sp096*** | 64 | 28 | <0.001 | 0.656 | 0.55-0.75 | 32.8 | 100 | 8.35x10-6 | 0.533 | ND | 0.67 | Poor |
| ***Leptotrichia sp212*** | 64 | 28 | <0.001 | 0.751 | 0.65-0.84 | 65.6 | 85.7 | 6.69x10-6 | 0.706 | 4.59 | 0.4 | Poor |
| ***Leptotrichia sp221*** | 64 | 28 | <0.001 | 0.757 | 0.66-0.84 | 56.2 | 92.9 | 8.35x10-6 | 0.674 | 7.88 | 0.47 | Poor |
| ***Leptotrichia sp223*** | 64 | 28 | 0.002 | 0.66 | 0.55-0.76 | 45.3 | 92.9 | 1.97x10-5 | 0.59 | 6.34 | 0.59 | Poor |
| ***Leptotrichia sp417*** | 64 | 28 | <0.001 | 0.763 | 0.66-0.85 | 67.2 | 89.3 | 4.56x10-5 | 0.728 | 6.27 | 0.37 | Poor |
| ***Leptotrichia sp498*** | 64 | 28 | <0.001 | 0.644 | 0.54-0.74 | 31.2 | 96.4 | 6.2x10-6 | 0.5 | 8.75 | 0.71 | Poor |
| ***Megasphaera micronuciformis*** | 64 | 28 | 0.005 | 0.67 | 0.57-0.77 | 75 | 57.1 | >0 | 0.6957 | 1.75 | 0.44 | Poor |
| ***Neisseria elongata*** | 64 | 28 | 0.005 | 0.64 | 0.54-0.74 | 45.3 | 89.3 | 1.4x10-5 | 0.565 | 4.23 | 0.61 | Poor |
| ***Neisseria oralis*** | 64 | 28 | <0.001 | 0.711 | 0.61-0.80 | 42.2 | 100 | >0 | 0.5978 | ND | 0.58 | Poor |
| ***Oribacterium parvum*** | 64 | 28 | <0.001 | 0.656 | 0.55-0.75 | 31.2 | 100 | >0 | 0523 | ND | 0.69 | Poor |
| ***Oribacterium sinus*** | 64 | 28 | <0.001 | 0.786 | 0.69-0.86 | 78.1 | 75 | 4.02x10-5 | 0.772 | 3.12 | 0.29 | Good/Poor |
| ***Porphyromonas pasteri*** | 64 | 28 | 0.006 | 0.655 | 0.55-0.75 | 35.9 | 96.4 | 1.13x10-3 | 0.544 | 10.1 | 0.66 | Poor |
| ***Prevotella histicola*** | 64 | 28 | <0.001 | 0.739 | 0.64-0.83 | 71.9 | 78.6 | 6.93x10-6 | 0.7283 | 3.35 | 0.36 | Poor |
| ***Ruminococcaceae G1 sp075*** | 64 | 28 | <0.001 | 0.809 | 0.71-0.88 | 70.3 | 92.9 | 8.35x10-6 | 0.772 | 9.84 | 0.32 | Poor |
| ***Selenomonas sp136*** | 64 | 28 | <0.001 | 0.682 | 0.35-0.58 | 50 | 85.7 | 6.17x10-6 | 0.598 | 3.5 | 0.58 | Poor |
| ***Stomatobaculum sp097*** | 64 | 28 | <0.001 | 0.735 | 0.63-0.82 | 56.2 | 89.3 | 3.99x10-5 | 0.652 | 5.25 | 0.49 | Poor |
| ***Tannerella sp286*** | 64 | 28 | 0.002 | 0.667 | 0.56-0.76 | 35.9 | 96.4 | 1.0x10-4 | 0.533 | 10.1 | 0.66 | Poor |
| ***TM7 G3 sp351*** | 64 | 28 | <0.001 | 0.67 | 0.56-0.76 | 31.2 | 100 | 6.79x10-5 | 0.641 | ND | 0.69 | Poor |
| ***TM7 G1 sp352*** | 64 | 28 | <0.001 | 0.792 | 0.69-0.87 | 56.2 | 92.9 | 4.93x10-5 | 0.533 | 7.88 | 0.47 | Good/Poor |
| ***Veillonella rogosae*** | 64 | 28 | <0.001 | 0.734 | 0.63-0.82 | 59.4 | 89.3 | 7.93x10-6 | 0.685 | 5.54 | 0.46 | Poor |

**(a)** Grp-All all samples ROC curve analysis

| **Grp-All Species^a^** | **# HPV+^b^** | **# HPV-^c^** | **p-value^d^** | **AUC^e^** | **95% CI^f^** | **Sensitivity^g^** | **Specificity^h^** | **Associated Criterion^i^** | **Accuracy^j^** | **LR+^k^** | **LR-^l^** | **Quality^m^** |
| --- | --- | --- | --- | --- | --- | --- | --- | --- | --- | --- | --- | --- |
| ***Fusobacterium periodonticum*** | 60 | 26 | <0.001 | 0.897 | 0.81-0.95 | 71.7 | 92.3 | 6.34x10^-4^ | 0.767 | 9.32 | 0.31 | Good |
| ***Gemella sanguinis*** | 62 | 25 | <0.001 | 0.764 | 0.66-0.85 | 88.7 | 56 | 5.35x10^-5^ | 0.782 | 2.02 | 0.2 | Good |
| ***Haemophilus parainfluenzae*** | 62 | 26 | <0.001 | 0.786 | 0.69-0.87 | 69.4 | 76.9 | 3.50x10^-3^ | 0.705 | 3.01 | 0.4 | Poor |
| ***Haemophilus pittmaniae*** | 48 | 8 | <0.001 | 0.927 | 0.83-0.98 | 72.9 | 100 | 1.08x10^-4^ | 0.768 | ND | 0.27 | Excellent/ Good |
| ***Lachnospiraceae G2 sp096*** | 23 | 2 | <0.001 | 0.957 | 0.79-0.99 | 91.3 | 100 | 8.35x10^-6^ | 0.920 | ND | 0.09 | Excellent |
| ***Leptotrichia sp221*** | 37 | 3 | <0.001 | 0.847 | 0.70-0.94 | 75.7 | 100 | 6.02x10^-6^ | 0.900 | ND | 0.24 | Excellent/ Good |
| ***Leptotrichia sp223*** | 36 | 11 | <0.001 | 0.841 | 0.71-0.93 | 80.6 | 81.8 | 1.97x10^-5^ | 0.787 | 4.43 | 0.24 | Good |
| ***Leptotrichia sp417*** | 49 | 16 | <0.001 | 0.879 | 0.77-0.95 | 87.8 | 81.2 | 4.56x10^-5^ | 0.846 | 4.68 | 0.15 | Good |
| ***Leptotrichia sp498*** | 22 | 2 | 0.006 | 0.878 | 0.61-0.94 | 72.7 | 100 | 1.80x10^-5^ | 0.708 | ND | 0.27 | Good |
| ***Neisseria elongate*** | 32 | 8 | 0.013 | 0.754 | 0.59-0.88 | 90.6 | 62.5 | 1.40x10^-5^ | 0.825 | 2.42 | 0.15 | Good |
| ***Oribacterium sinus*** | 57 | 16 | 0.001 | 0.748 | 0.63-0.84 | 87.7 | 56.2 | 4.02x10^-5^ | 0.808 | 2.01 | 0.22 | Good/Poor |
| ***Porphyromonas pasteri*** | 52 | 22 | <0.001 | 0.721 | 0.61-0.82 | 44.2 | 95.5 | 1.13x10^-3^ | 0.595 | 9.73 | 0.58 | Poor |
| ***Ruminococcaceae G1 sp075*** | 46 | 5 | 0.034 | 0.804 | 0.67-0.90 | 78.3 | 80 | 3.78x10^-5^ | 0.765 | 3.91 | 0.27 | Good/Poor |
| ***Tannerella sp286*** | 40 | 11 | 0.004 | 0.707 | 0.56-0.83 | 50 | 100 | 1.06x10^-4^ | 0.588 | ND | 0.5 | Poor |
| ***TM7 G3 sp351*** | 47 | 7 | <0.001 | 0.8 | 0.63-0.92 | 66.7 | 100 | 6.79x10^-5^ | 0.704 | 4.91 | 0.35 | Good/Poor |
| ***TM7 G1 sp352*** | 30 | 5 | <0.001 | 0.769 | 0.63-0.87 | 70.2 | 85.7 | 1.00x10^-5^ | 0.800 | ND | 0.33 | Good |
| ***Veillonella rogosae*** | 41 | 7 | 0.036 | 0.742 | 0.59-0.86 | 92.7 | 57.1 | 7.93x10^-6^ | 0.875 | 2.16 | 0.13 | Good/Poor |

**(b)** Grp-All zeros minimized ROC analysis

| **Grp-noAB Species^a^** | **# HPV+^b^** | **# HPV-^c^** | **p-value^d^** | **AUC^e^** | **95% CI^f^** | **Sensitivity^g^** | **Specificity^h^** | **Associated Criterion^i^** | **Accuracy^j^** | | **LR+^k^** | **LR-^l^** | **Quality^m^** |
| --- | --- | --- | --- | --- | --- | --- | --- | --- | --- | --- | --- | --- | --- |
| ***Actinomyces sp172*** | 48 | 12 | 0.003 | 0.703 | 0.57-0.81 | 54.2 | 91.7 | 9.1x10-6 | | 0.617 | 6.5 | 0.50 | Poor |
| ***Alloprevotella sp473*** | 48 | 12 | <0.001 | 0.708 | 0.58-0.82 | 41.7 | 100 | >0 | | 0.533 | ND | 0.58 | Poor |
| ***Alloprevotella tannerae*** | 48 | 12 | 0.005 | 0.701 | 0.57-0.81 | 60.4 | 83.3 | >0 | | 0.65 | 1.94 | 0.53 | Poor |
| ***Capnocytophaga sputigena*** | 48 | 12 | <0.001 | 0.735 | 0.61-0.84 | 45.8 | 100 | 9.0x10-6 | | 0.55 | ND | 0.54 | Poor |
| ***Catonella morbi*** | 48 | 12 | 0.001 | 0.731 | 0.60-0.83 | 60.4 | 83.3 | 1.59x10-5 | | 0.633 | 3.63 | 0.48 | Poor |
| ***Fusobacterium periodonticum*** | 48 | 12 | <0.001 | 0.877 | 0.77-0.95 | 77.1 | 100 | 6.34x10-4 | | 0.8 | ND | 0.23 | Good |
| ***Gemella sanguinis*** | 48 | 12 | <0.001 | 0.804 | 0.68-0.90 | 93.7 | 58.3 | 5.36x10-5 | | 0.933 | 2.25 | 0.11 | Good |
| ***Haemophilus parainfluenzae*** | 48 | 12 | 0.001 | 0.747 | 0.62-0.85 | 41.7 | 100 | 1.69x10-2 | | 0.533 | ND | 0.58 | Poor |
| ***Haemophilus pittmaniae*** | 48 | 12 | <0.001 | 0.884 | 0.76-0.95 | 83.3 | 91.7 | 1.81x10-5 | | 0.833 | 10 | 0.18 | Good |
| ***Lachnoanaerobaculum orale*** | 48 | 12 | 0.003 | 0.748 | 0.62-0.85 | 70.8 | 83.3 | 2.38x10-5 | | 0.733 | 4.25 | 0.35 | Poor |
| ***Lachnospiraceae G3 sp100*** | 48 | 12 | <0.001 | 0.698 | 0.57-0.81 | 39.6 | 100 | >0 | | 0.517 | ND | 0.60 | Poor |
| ***Leptotrichia sp212*** | 48 | 12 | <0.001 | 0.806 | 0.68-0.90 | 62.5 | 100 | 6.69x10-6 | | 0.683 | ND | 0.38 | Poor |
| ***Leptotrichia sp215*** | 48 | 12 | <0.001 | 0.901 | 0.80-0.96 | 81.2 | 100 | 1.8x10-5 | | 0.833 | ND | 0.19 | Excellent/ Good |
| ***Leptotrichia sp221*** | 48 | 12 | <0.001 | 0.794 | 0.67-0.89 | 64.6 | 91.7 | >0 | | 0.7 | 7.75 | 0.39 | Good/Poor |
| ***Leptotrichia sp392*** | 48 | 12 | <0.001 | 0.729 | 0.60-0.84 | 52.1 | 100 | 9.07x10-6 | | 0.617 | ND | 0.48 | Poor |
| ***Leptotrichia sp417*** | 48 | 12 | <0.001 | 0.738 | 0.61-0.84 | 66.7 | 100 | 1.4x10-5 | | 0.717 | ND | 0.33 | Poor |
| ***Leptotrichia sp879*** | 48 | 12 | <0.001 | 0.688 | 0.56-0.80 | 37.5 | 100 | >0 | | 0.5 | ND | 0.63 | Poor |
| ***Megasphaera micronuciformis*** | 48 | 12 | 0.007 | 0.707 | 0.58-0.82 | 58.3 | 83.3 | 2.68x10-5 | | 0.633 | 3.5 | 0.5 | Poor |
| ***Neisseria oralis*** | 48 | 12 | <0.001 | 0.698 | 0.57-0.81 | 39.6 | 100 | >0 | | 0.517 | ND | 0.6 | Poor |
| ***Oribacterium parvum*** | 48 | 12 | <0.001 | 0.698 | 0.57-0.81 | 39.6 | 100 | >0 | | 0.517 | ND | 0.6 | Poor |
| ***Oribacterium sinus*** | 48 | 12 | 0.001 | 0.741 | 0.61-0.85 | 77.1 | 66.7 | 4.02x10-5 | | 0.75 | 2.31 | 0.34 | Poor |
| ***Prevotella histicola*** | 48 | 12 | <0.001 | 0.854 | 0.74-0.93 | 70.8 | 100 | >0 | | 0.767 | ND | 0.29 | Good |
| ***Prevotella nanceiensis*** | 48 | 12 | 0.003 | 0.711 | 0.58-0.82 | 64.6 | 83.3 | 3.20x10-5 | | 0.683 | 3.88 | 0.43 | Poor |
| ***Prevotella pallens*** | 48 | 12 | 0.003 | 0.705 | 0.57-0.82 | 62.5 | 83.3 | 1.34x10-5 | | 0.667 | 3.75 | 0.45 | Poor |
| ***Ruminococcaceae G1 sp075*** | 48 | 12 | <0.001 | 0.866 | 0.75-0.94 | 77.1 | 100 | 4.55x10-6 | | 0.817 | ND | 0.23 | Good |
| ***Selenomonas sp136*** | 48 | 12 | <0.001 | 0.76 | 0.63-0.86 | 52.1 | 100 | >0 | | 0.617 | ND | 0.48 | Poor |
| ***TM7 G1 sp352*** | 48 | 12 | <0.001 | 0.807 | 0.69-0.90 | 56.25 | 100 | 1.0x10-5 | | 0.733 | ND | 0.44 | Good/Poor |
| ***TM7 G3 sp351*** | 48 | 12 | <0.001 | 0.736 | 0.61-0.84 | 47.9 | 100 | 9.0x10-6 | | 0.567 | ND | 0.52 | Poor |
| ***Veillonella rogosae*** | 48 | 12 | <0.001 | 0.8 | 0.68-0.89 | 68.7 | 91.7 | 7.93x10-6 | | 0.733 | 8.25 | 0.34 | Good/Poor |

**(c)** Grp-noAB all samples ROC analysis

**(d)** Grp-noAB zeros minimized ROC analysis

| **Grp-noAB Species^a^** | **# HPV+^b^** | **# HPV-^c^** | **p-value^d^** | **AUC^e^** | **95% CI^f^** | **Sensitivity^g^** | **Specificity^h^** | **Associated Criterion^i^** | **Accuracy^j^** | **LR+^k^** | **LR-^l^** | **Quality^m^** |
| --- | --- | --- | --- | --- | --- | --- | --- | --- | --- | --- | --- | --- |
| ***Actinomyces sp173*** | 32 | 5 | 0.004 | 0.781 | 0.62-0.90 | 81.2 | 80 | 9.1x10-6 | 0.729 | 4.06 | 0.23 | Poor |
| ***Catonella morbi*** | 33 | 4 | 0.047 | 0.735 | 0.54-0.87 | 45.5 | 100 | 2.8x10-4 | 0.786 | ND | 0.55 | Poor |
| ***Fusobacterium periodonticum*** | 44 | 12 | <0.001 | 0.956 | 0.87-0.99 | 84.1 | 100 | 6.34x10-4 | 0.857 | ND | 0.16 | Excellent/ Good |
| ***Gemella sanguinis*** | 47 | 12 | <0.001 | 0.821 | 0.70-0.91 | 95.7 | 58.3 | 5.35x10-5 | 0.864 | 2.3 | 0.07 | Good |
| ***Haemophilus parainfluenzae*** | 47 | 12 | 0.001 | 0.762 | 0.63-0.86 | 42.6 | 100 | 1.69x10-2 | 0.542 | ND | 0.57 | Poor |
| ***Haemophilus pittmaniae*** | 42 | 5 | <0.001 | 0.924 | 081-0.98 | 95.2 | 80 | 1.81x10-5 | 0.915 | 4.76 | 0.06 | Excellent |
| ***Leptotrichia sp215*** | 40 | 2 | <0.001 | 0.988 | 0.89-1.0 | 97.5 | 100 | 1.80x10-5 | 0.952 | ND | 0.03 | Excellent |
| ***Leptotrichia sp392*** | 26 | 2 | <0.001 | 0.962 | 0.81-0.99 | 96.2 | 100 | 9.1x10-6 | 0.964 | ND | 0.04 | Excellent |
| ***Leptotrichia sp417*** | 33 | 8 | <0.001 | 0.996 | 0.91-1.0 | 97 | 100 | 1.4x10-5 | 0.951 | ND | 0.03 | Excellent |
| ***Oribacterium sinus*** | 42 | 9 | 0.001 | 0.772 | 0.63-0.89 | 69 | 77.8 | 8.64x10-4 | 0.686 | 3.11 | 0.4 | Poor |
| ***Prevotella nanceiensis*** | 33 | 5 | 0.039 | 0.764 | 0.60-0.87 | 93.9 | 60 | 3.19x10-5 | 0.868 | 2.35 | 0.1 | Good |
| ***Prevotella pallens*** | 34 | 6 | 0.001 | 0.784 | 0.63-0.90 | 55.9 | 100 | 4.06x10-4 | 0.625 | ND | 0.44 | Poor |
| ***Ruminococcaceae G1 sp075*** | 37 | 2 | <0.001 | 1 | 0.91-1.0 | 100 | 100 | 4.55x10-6 | 1 | ND | 0 | Excellent |
| ***TM7 G1 sp352*** | 27 | 4 | <0.001 | 0.845 | 0.70-0.94 | 73 | 100 | 7.0x10-5 | 0.732 | ND | 0.27 | Poor |
| ***TM7 G3 sp351*** | 34 | 2 | <0.001 | 0.907 | 0.74-0.98 | 85.2 | 100 | 9.0x10-6 | 0.827 | ND | 0.15 | Excellent/ Good |

Summary statistics of significant Receiver Operating Characteristic (ROC) curve analysis comparing HPV-positive (HPV+) patient samples to HPV-negative (HPV-) patient samples of **(A)** Grp-All, **(B)** Grp-All zeros inflation minimized, **(C)** Grp-noAB, and **(D)** Grp-noAB zero inflation minimized for sample site combination of buccal, plaque, saliva, and tongue (BPST) samples.

**^a^**Species counts transformed using the formula log(RA+1) and excluding zeros from analysis.

**^b^**The number of HPV+ samples.

**^c^**The number of HPV- samples.

**^d^**The probability that the observed sample area under the ROC curve is found when the true population is 0.5.

**^e^**The area under the curve (AUC).

**^f^**The 95 percent confidence interval.

**^g^**The probability that a test result will be positive when the species is present.

**^h^**The probability that a test result will be negative when the species is not present.

**^i^**The Youden’s Index associated criterion value used as the optimum cut-off point for a diagnostic test.

**^j^**Accuracy calculated as the number of present and positive divided by the number of present and positive plus the number of present and negative plus the number of absent and positive plus the number of absent and negative ((a+d)/(a+b+c+d)), as described by Ray et al., 2010.

**^k^**Positive likelihood ratio.

**^l^**Negative likelihood ratio.

**^m^**Overall quality of the species as a biomarker determined, as described by Ray et al., 2010.
