# Supplementary material for: Haemophilus pittmaniae and Leptotrichia spp. Constitute a Multi-Marker Signature in a Cohort of Human Papillomavirus-Positive Head and Neck Cancer Patients
Source: Front Microbiol. 2022 Jan 18;12:794546. doi: 10.3389/fmicb.2021.794546 (PMC8803733; doi:10.3389/fmicb.2021.794546)
Supplement: Supplementary file 7 [file Data_Sheet_3.docx]

**Figure S1.** PCoA plots of Grp-All comparisons for the BPST sample site combination

**A)** HNC *vs.* HC BPST

**
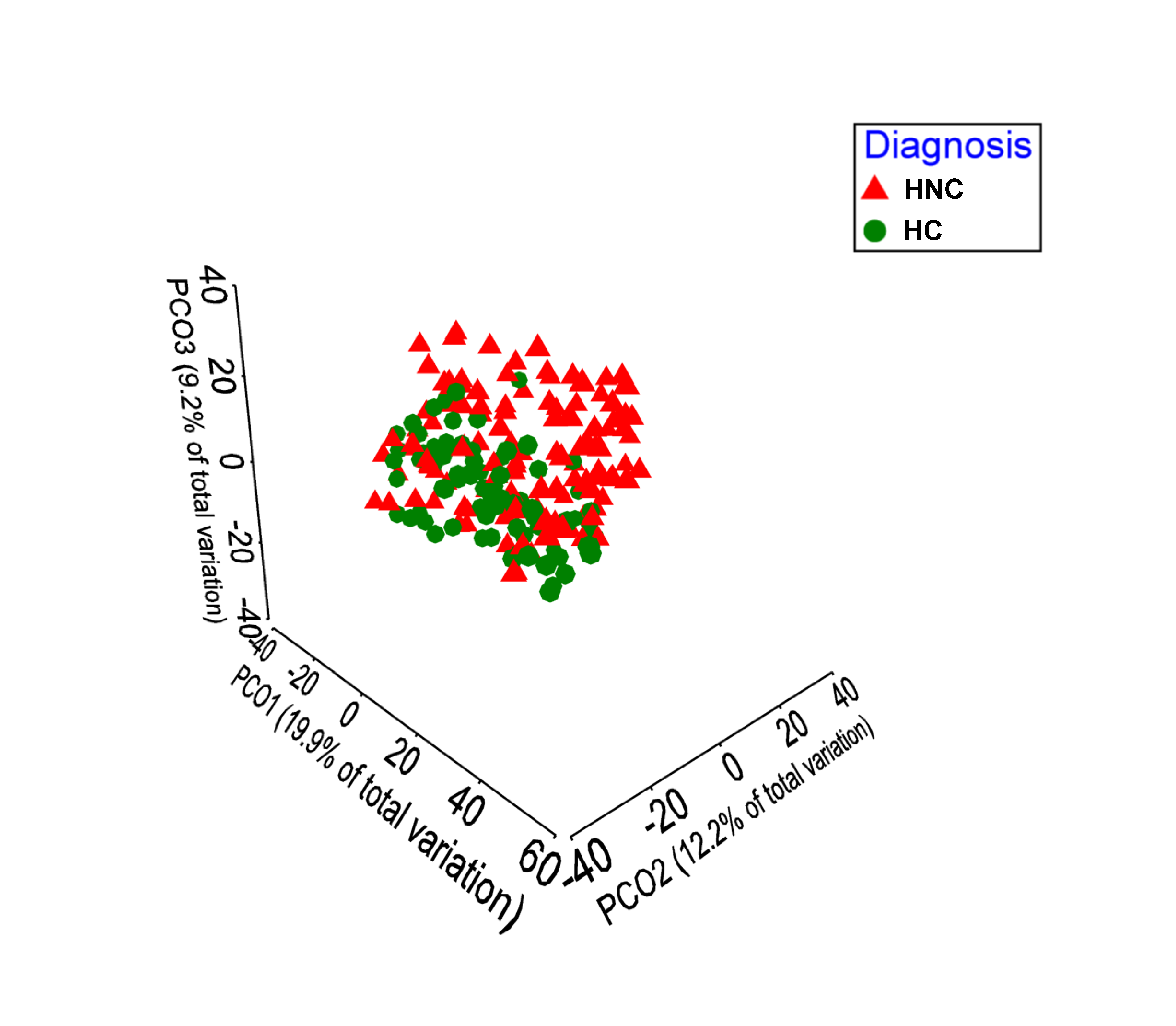
**


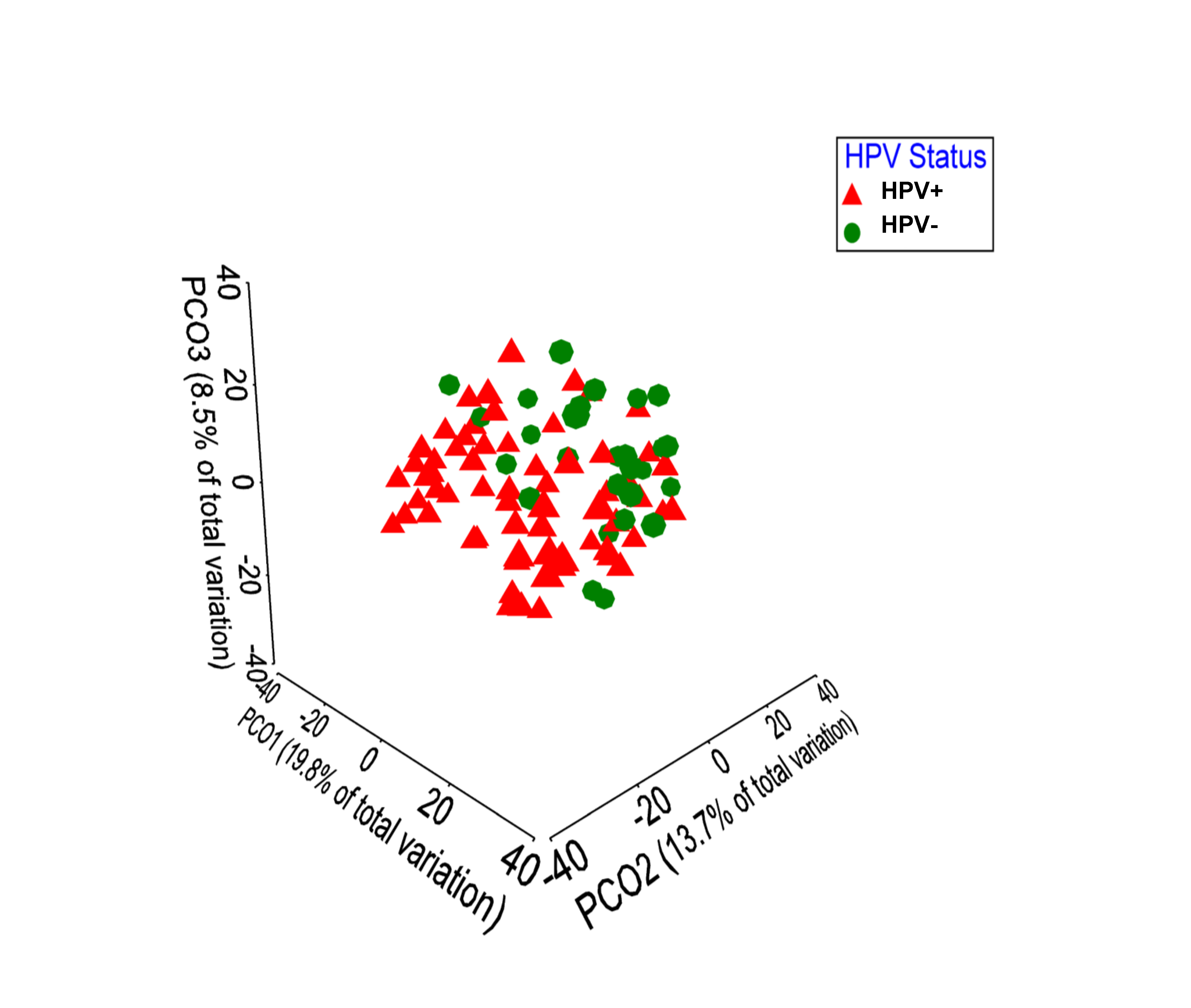
**B)** HPV+ *vs.* HPV- BPST

**Legend.**

Principal coordinate analysis (PCoA) was completed for Grp-All (**A**) HNC *vs.* HC for ‘Diagnosis’ and (**B**) HPV+ *vs.* HPV- for ‘HPV Status’ BPST sample comparisons using squared-root transformed relative abundance data converted into Bray-Curtis similarity matrices.
